# Methodological approaches and author-reported limitations in evaluation studies of digital health technologies (DHT): A scoping review of DHT interventions for cancer, diabetes mellitus, and cardiovascular diseases

**DOI:** 10.1371/journal.pdig.0000806

**Published:** 2025-04-24

**Authors:** Nyangi Gityamwi, Jo Armes, Jenny Harris, Emma Ream, Richard Green, Anand Ahankari, Alison Callwood, Athena Ip, Jane Cockle-Hearne, Wendy Grosvenor, Agnieszka Lemanska, Simon S. Skene

**Affiliations:** 1 School of Health Sciences, University of Surrey, Guildford, Surrey, United Kingdom; 2 NIHR Applied Research Collaboration—Kent, Surrey and Sussex, Sussex Partnership NHS Foundation Trust, Hove, United Kingdom; 3 School of Biosciences, University of Surrey, Guildford, Surrey, United Kingdom; Iran University of Medical Sciences, IRAN, ISLAMIC REPUBLIC OF

## Abstract

Digital health technology (DHT) holds the potential to improve health services, and its adoption has proliferated in recent decades owing to technological advancement. Optimal evaluation methodologies appropriate for generating quality evidence on DHT have yet to be established; traditional comparative designs present several limitations. This study aimed to scope the literature to highlight common methodological approaches used and their limitations to inform considerations for designing robust DHT evaluation studies. A scoping review was conducted following the Joanna Briggs Institute (JBI) scoping review guidelines. A systematic search was conducted using the CINAHL (EBSCO), MEDLINE (EBSCOhost), PsycINFO (EBSCO), EMBASE (Elsevier) and Web of Science (Clarivate Analytics) databases using iteratively developed search terms. We selected studies published in English between January 2016 and March 2022 and focussed on primary research evaluating the effectiveness of DHT with technology-user interactive or asynchronous features for adults (≥18 years) with cancer, diabetes or cardiovascular conditions. The final number of articles, after the screening and selection process, comprised 140 records. Data were analysed descriptively (frequency and percentages) and summarised thematically. Results showed most studies (*n* = 104, 74.3%) employed the standard two-arm parallel RCT design, with usual/standard care as the preferred comparator in nearly half (*n* = 65, 47.1%) of all included studies. Of the 104 comparative studies reviewed, limitations in *recruitment* were most frequently reported (*n* = 70, 37%), followed by limitations in *evaluation/measurement techniques* (*n* = 57, 27%), presence of *confounding factors* (*n* = 50, 24%) and *short duration of studies* (*n* = 24, 11%). The review highlights the need to consider inclusive approaches to recruitment and adoption of the emerging methodological approaches that account for the fast-paced, multi-component and group contamination problem resulting from the unconcealable nature of DHT interventions.

## Author summary

There is growing potential for digital health technology to improve healthcare delivery and access. However, digital health interventions are usually complex creating methodological challenges in their evaluation. In this article, we present findings of a scoping review on common methodological approaches and limitations and/or issues in studies evaluating digital health interventions for three common long-term conditions (i.e., diabetes, cancer and cardiovascular disease). Our findings suggest that the standard, two-arm parallel randomised controlled trials with usual/standard care as comparator, remain the preferred methodological approach. The common methodological limitation reported in the comparative study designs was bias in selection and recruitment, either due to biased eligibility criteria or recruiting highly motivated and technology-savvy participants. Other issues reported are the unreliability of the measurement techniques used, and inability of investigators to fully control the environment in which the digital health intervention was tested and/or control participants’ access to the intervention. This reduces the generalisability and credibility of the studies’ findings. Based on our findings, researchers need to advertise their studies widely using varied communication channels and provide support to less technical participants to ensure the recruitment of a diverse and representative sample. Also, there is a need to consider emerging methodological approaches that allow multi-phase and multiple-group comparisons in an attempt to address these limitations.

## Introduction

Digital health technology (DHT) has the potential to improve health and social care by widening access for individuals [1]. DHT has sparked public interest as it can significantly help increase patients’ engagement, empower them to manage their conditions [2] and ensure continuity of care, as was the case during the COVID-19 pandemic lockdowns.

Nevertheless, as with any healthcare service or product, the use of DHT needs to be supported by robust evidence to show it is accurate, reliable, safe and able to demonstrate effectiveness in producing the intended outcome [2]. Despite the potential of DHT interventions, evaluation faces challenges due to their complex nature and evolving technology. The randomisation approaches and tight control of the research environment [3] make randomised controlled trials (RCTs) the accepted gold standard methodology for the unbiased demonstration of causal relationships between intervention and outcome [4]. Consequently, RCTs have been widely employed when testing digital health interventions [5,6]. RCTs are both recommended in the World Health Organisation’s (WHO) practical guideline for monitoring and evaluating digital health interventions [7] and endorsed by the National Institute for Health & Care Excellence (NICE) [2].

However, since RCT protocols require a static and tightly controlled environment to minimise bias and confounding factors [3], this poses challenges regarding the appropriateness of RCTs as an evaluation design for DHT because of their continuous technological evolution [8]. Moreover, long timeframes required for RCTs [9] mean a DHT may be outdated when the evaluation of its efficiency has been completed. Therefore, the utility of RCTs to evaluate the complex and multifaceted nature of DHT interventions is a subject of debate [10], particularly when the technology is to be scaled up and tested in real-world complex health systems. Instead, methodological approaches that allow flexibility and context-dependent evaluation are gaining importance [11,12]. The UK Medical Research Council has proposed a range of other designs that can be used as an alternative to the standard RCT such as stepped-wedge or N-of-1 designs [13]. These and other designs can provide greater flexibility regarding measurements and their timing, dosage and length of intervention/exposure duration [14,15]. Examples include the sequential multiple assignment randomised trial (SMART) and multiphase optimisation strategy (MOST), including factorial designs to measure effects as presented by Collins and colleagues [16]. Indeed, few studies have used adaptive study designs for the evaluation of DHTs, as noted by both Pham and colleagues [17] and the more recent scoping review by Hrynyschyn and colleagues [18] of designs used other than RCTs. Our scoping review aimed to contribute to this body of literature by reviewing and critically analysing practical implications of the comparative study designs, highlighting limitations and issues from the researchers’ reported experience. We aimed to answer two research questions:

i. What study designs are used to evaluate the impact of DHT on clinical health outcomes among adults diagnosed with diabetes, cancer and cardiovascular diseases?
ii. What are the author-reported methodological challenges in comparative study designs evaluating DHT?

## Methods

This review was conducted in accordance with the Joanna Briggs Institute (JBI) scoping review guidelines [19]. Scoping reviews are useful for examining emerging and complex fields of study and when the research focus or question is broad [20,21]; thus, very relevant for systematically collating research evidence with varied study designs used in the evaluation of digital health interventions. The protocol is registered with Open Science Framework (OSF) https://doi.org/10.17605/OSF.IO/R24D5.

This paper reports the findings from the scoping review by following the reporting guidelines of the PRISMA-ScR (Preferred Reporting Items for Systematic Reviews and Meta-Analyses Extension for Scoping Reviews) checklist [22].

### Eligibility and inclusion criteria

The review was conducted at a time of high proliferation of DHT and research studies evaluating them were numerous and heterogeneous in terms of their focus. To address this, our review focussed on studies conducted to evaluate the impact of digital health interventions on three long-term conditions namely diabetes, cancer and cardiovascular disease. These conditions were selected due to their chronic nature, and the significant burden they place on individuals and the healthcare system globally [23]. Additionally, their importance in the digital health landscape was a key factor in their selection [24]. Furthermore, only studies that evaluated the effectiveness or efficacy of DHT interventions were eligible for inclusion in the final data synthesis. We excluded proof of concept studies and those which only assessed acceptability or usability without clinical or treatment outcomes. Table 1 presents the inclusion and exclusion criteria that were applied in the study screening and selection.

**Table 1:** A summary of study inclusion and exclusion criteria used.

Criteria type	Inclusion criteria	Exclusion criteria
Publication type	Peer-reviewed primary research	Protocols, reviews, editorials, commentaries
Language	Publications in English language	Non- English language publications
Publication age	January 2016–March 2022	Published before January 2016 or after March 2022
Age of study participants	Adults ≥ 18 years	Individuals aged less than 18 years
Conditions investigated	Cancer (any type), Diabetes mellitus, Cardiovascular disease	Other long-term conditions
DHT type	With technology-user interaction or asynchronous features, e.g., disease self-monitoring or self-management solutions, patient - care provider consultation, On-demand health information for clients, etc.	Non-interactive or synchronised DHT such as emails, phone calls, video recordings or text messages
Outcome evaluated	Clinical outcome measures	Usability, acceptability, proof of concepts

Because of the breadth of DHT, we narrowed the review to include only those DHTs with technology-user interactive (asynchronous) features. The search was limited to articles published from January 2016 with the last search conducted in March 2022. This time frame was selected to capture the recent studies technology at the time of the review.

### Information sources and search strategy

An initial limited search was undertaken in MEDLINE (EBSCOhost) in consultation with a research librarian to identify the database-specific index terms related to two key concepts—“digital health” and “disease management”. The search strategy was then adapted for each included database and first search was performed between March and June 2020 in CINAHL (EBSCO), MEDLINE (EBSCOhost), PsycINFO (EBSCO), EMBASE (Elsevier) and Web of Science (Clarivate Analytics). In March 2022, we conducted updated search in EBSCOhost, a collection of MEDLINE, CINAHL and PsycINFO databases (Table 2). The complete search strategies and results for both searches are provided in S1 File.

**Table 2:** Scoping review search query used for MEDLINE(EBSCOhost).

Database	Search query	Limiters
EBSCOhost search (Medline + CINAHL + PsycINFO)	SU (Telemedicine OR telerehabilitation OR “remote consultation” OR “internet-based intervention” OR “mobile application” OR smartphone OR “cell phone” OR “Therapy, computer-assisted”) AND SU (“delivery of health care” OR “health” OR “health promotion” OR “disease management” OR “treatment outcome” OR therapeutics OR “rehabilitation OR “patient care” OR nursing) AND SU (diabetes OR cancer OR hypertension OR cardiovascular)	*Published Date*: 2020/05/01–2022/03/31.
		*English Language*
		Peer Reviewed; Research Article.
		Age-Related: All Adults: 19+ years;
		**Expanders** Apply related words; Apply equivalent subjects
		**Search modes** Boolean/Phrase

### Reference screening and selection

**Fig 1:**
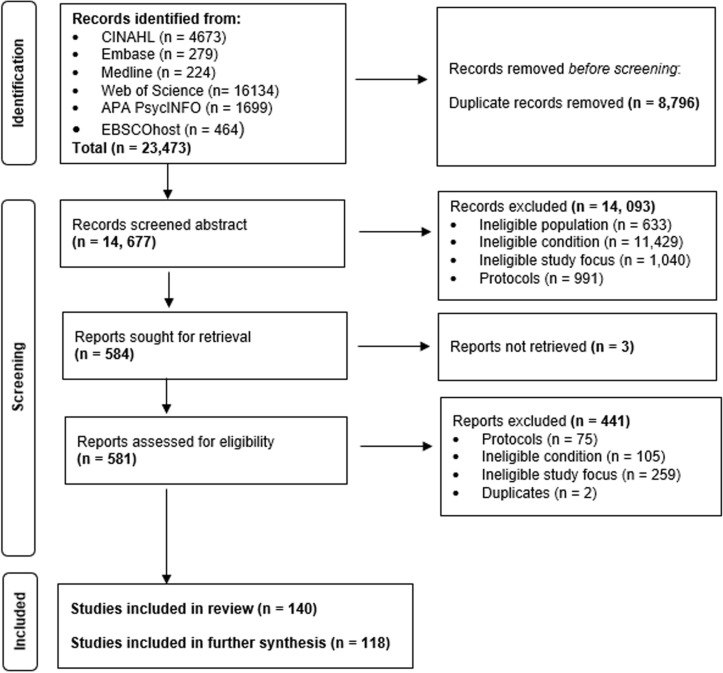
Preferred Reporting Items for Systematic Reviews and Meta-Analyses (PRISMA) flowchart depicting the process of identifying and screening publications included in the scoping review.

All identified citations were retrieved and imported into the reference manager software EndNote^X9^ (Clarivate Analytics, PA, USA). Duplicates were removed by the lead researcher and author (NG). All titles and abstracts were screened independently for eligibility against the inclusion and exclusion criteria by NG, and then a random sub-sample of 20% of papers was reviewed by other members of the research team (SS, JA, JH) to ensure consistency in applying the eligibility criteria. Where there were discrepancies in study selection, full articles were collectively reviewed by members of the research team and discussed to reach a consensus. The full text of eligible citations was then assessed in detail against the inclusion criteria by two reviewers independently (NG and JH). Any disagreements that arose between the reviewers were resolved through discussion with a third (JA or SS). Fig 1 shows the study selection process using the Preferred Reporting Items for Systematic Reviews and Meta-Analyses extension for scoping reviews (PRISMA-ScR). The description of studies included in the review and data extracted is presented in S2 File.

### Data charting

Data charting guide was created in Microsoft Excel by the primary author (NG) and then discussed and verified by two co-authors (JA and SS). Pilot data charting (20% of the included articles) was performed by three investigators independently (JH, JA and ER) to test the guide, any discrepancies were discussed until consensus was reached and the guide was revised as appropriate. The final full data charting guide (presented in S3 File) includes but is not limited to a description of the references, condition investigated, aim of the digital health intervention, DHT classification, study design used and comments on methodology. All investigators participated in data extraction using a Microsoft Excel spreadsheet.

### Data synthesis

Data were extracted from 140 articles and the initial analysis was performed to broadly describe the characteristics of the included studies and study designs adopted to evaluate DHT for the selected long-term conditions (research question 1). Of these studies, 118 studies were controlled/comparative studies, and these were selected to summarise author-reported methodological challenges (research question 2). Data were presented in percentages in general and according to the condition investigated and categories of the DHT aims. The NICE Evidence Standards Framework [25] for digital health technologies was used to define and classify DHTs according to their aim. We reported the challenges in the context of the author’s reported limitations.

## Results

### Search results

The two searches identified a total of 23,473 records. Following the removal of duplicates (*n* = 8,796), the titles and abstracts of 14,677 articles were screened and 584 were selected for full-text review. A further 444 articles were excluded following the full-text review with the reasons outlined (Fig 1) and the final articles included were 140.

### Characteristics of the reviewed references

Of the 140 articles, 11 (7.9%) were published in 2016, 21 (15%) in 2017, 19 (13.6%) in 2018, 15 (10.7%) in 2019, 42 (30%) in 2020, 30 (21.4%) in 2021, with only 2 (1.4%) published in 2022. Studies were most frequently conducted in the United States of America (*n* = 29, 20.7%) followed by the Netherlands and China each with 12 (8.6%) publications. Of the three included conditions, more than a third of the reviewed studies focussed on diabetes mellitus (*n* = 52, 37.1%). When grouped studies into three categories according to the aim of the DH interventions following the NICE classification framework [25], those aimed at providing treatment and/or therapies were 42.9%, (*n* = 60); aiding disease self-management were 35.7% (*n* = 50), and those aimed at promoting preventive behavioural change 21.4% (*n* = 30).

### Study designs used to evaluate the impact of DHT on clinical health outcomes

Six study designs were identified from the reviewed studies. These are RCTs; controlled, non-randomised quasi-experimental studies; pre-post single-arm follow-up; cross-sectional observation; mixed methods; and retrospective studies. Generally, RCTs were the most common research design, adopted in 104 of the 140 (74.3%) included studies (Table 3). This was the case regardless of the condition as we found RCTs comprised 76.2% of the cancer studies, 63.5% of the diabetes mellitus, 84.2% of the cardiovascular, and 87.5% of the studies with multiple conditions (Table 3). When classified according to intervention aim (i.e., DHT for *treatment/therapy, self-management, preventive/behavioural change*), RCTs were still the most commonly design, found in more than half of studies in all three groups.

**Table 3:** Numbers of articles included in the review, presented according to the disease condition, the aim of digital health intervention, study design, and type of comparator.

	Cancer *N* (%)	Diabetes mellitus *N* (%)	Cardiovascular *N* (%)	Multiple conditions *N* (%)	Total *N* (%)
	42 (30)	52 (37.1)	38 (27.1)	8 (5.7)	140 (100)
**Aim of DH intervention**					
*Treatment/therapy*	22 (52.3)	21 (40.4)	16 (42.1)	1 (12.5)	60 (42.9)
*Self-management*	11 (26.2)	25 (48.1)	10 (26.3)	4 (50.0)	50 (35.7)
*Preventive/behavioural change*	9 (21.4)	6 (11.5)	12 (31.6)	3 (37.5)	30 (21.4)
**Study design**					
*Randomised controlled trials*	32 (76.2)	33 (63.5)	32 (84.2)	7 (87.5)	104 (74.3)
*Controlled, non-randomised*	6 (14.3)	5 (9.6)	3 (7.9)	0	14 (10.7)
*Pre-post single-arm follow-up*	4 (9.5)	8 (15.3)	1 (2.6)	1	14 (9.3)
*Cross-sectional observation*	0	1 (1.9)	0	0	1 (0.7)
*Retrospective*	0	4 (7.7)	1 (2.6)	0	5 (3.6)
*Mixed methods*	0	1 (1.9)	1 (2.6)	0	2 (1.4)
**Type of comparator**					
*Usual care*	15 (35.7)	23 (46.2)	21 (55.3)	6 (75.0)	65 (47.1)
*Waitlist/delayed treatment*	11 (23.8)	3 (5.8)	1 (2.6)	0	15 (10)
*Active control/alternative intervention*	8 (19)	9 (19.2)	11 (28.9)	1 (12.5)	29 (21.4)
*Historic control*	4 (9.5)	3 (5.8)	3 (7.9)	0	10 (7.1)
*Non-responders*	0	1 (1.9)	0	0	1 (0.7)

Other study designs adopted included pre-post/prospective single-arm follow-ups (*n* = 14), retrospective studies (*n* = 5), mixed methods (*n* = 2), and a cross-sectional observational study (*n* = 1) (Table 3). Of the 14 pre-post studies, 9 evaluated DHT aimed at providing treatment/therapies, 4 evaluated DHT for patient self-management, and 1 study evaluated DHT for preventive/behavioural change. All five retrospective studies evaluated DHT for treatment/therapy. Of the two mixed-methods studies, one evaluated DHT for treatment/therapy, and the other evaluated DHT for preventive/behavioural change. The cross-sectional evaluation study evaluated DHT aimed at aiding patient self-management.

### Study designs according to health condition

#### Study designs used in cancer studies.

Of the 42 cancer-focussed studies, 76.2% (*n* = 32) adopted RCT designs, whereas 14.2% (*n* = 6) were non-randomised studies with controls and 9.5% (*n* = 4) were pre-post single-arm studies. More than half of the cancer studies which used RCT designs (63%, *n* = 20) were interventions aimed at providing *treatment/therapies*.

Other RCTs focussed on *patients’ self-management (31%, n = 10)* and *preventive/behavioural change (25%, n = 8).* Of the six non-randomised cancer studies, three focussed on *self-management* and the remaining three were studies aimed at providing *treatment/therapies.* Of the four pre- and post-studies, two focussed on *treatment/therapies*, one on *self-management*, and one on *preventive/behavioural change* category.

#### Study designs used in studies on diabetes mellitus.

Of the 52 studies involving patients with diabetes mellitus, 63.5% (*n* = 33) adopted RCT designs, whereas 9.6% (*n* = 5) were non-randomised studies with controls and 15.3% (*n* = 8) were pre-post single-arm studies. Additionally, there was one cross-sectional study, one study that employed a mixed methods design and four retrospective studies.

More than half of diabetes studies which adopted RCT designs (52%, *n* = 17) were interventions aimed at aiding *self-management*. Those that provided *treatment/therapies* numbered 10 (30%) while *preventive/behavioural change* interventions constituted 19.4% (*n* = 6) of the diabetes RCTs.

All five non-randomised studies with controls targeted self-management. Of the eight studies which employed a pre-post design, six were categorised as *disease treatment/therapy* whereas the remaining two were *preventive/behavioural change* interventions. All four retrospective studies focussed on providing *treatment/therapies.* The study that employed a cross-sectional design was a *self-management* intervention while the mixed-method study aimed to *provid*e *treatment/therapy.*

#### Study designs used in studies for cardiovascular diseases.

Of the 38 cardiovascular disease studies, 84.2% (*n* = 32) adopted RCT designs, whereas 7.9% (*n* = 3) were non-randomised studies with controls. Finally, there was a pre-post single-arm trial, a retrospective study, and one with a mixed method.

Of the 32 studies that adopted RCT designs, 41% (*n* = 13) evaluated interventions for *treatment/therapy*, 34% (*n* = 11) studies investigated *preventive/behavioural change* interventions and the remaining 25% (*n* = 8) evaluated *self-management interventions*. Of the three cardiovascular non-RCTs, two evaluated *treatment/therapies* whereas one evaluated a *self-management* intervention. The study which employed a pre-post single arm evaluated *self-management* intervention, the retrospective study evaluated a *treatment/therapies* intervention, and the mixed methods study evaluated a *preventive/behavioural change* intervention.

#### Study designs used in studies with multiple conditions.

Eight studies involved patients with multiple conditions. Seven of these adopted RCT designs (88%), whereas one employed a pre-post single-arm design (12%). Of the seven RCTs, four (57.1%) evaluated interventions for *self-management* and the remaining three studies evaluated *preventive/behavioural change* interventions. The pre-post study evaluated a treatment/therapy intervention.

### Comparative designs used in studies evaluating the effectiveness of DHT interventions

To address the question regarding the appropriateness of standard recommended comparative approaches such as RCTs for digital health evaluation, the present review presents limitations reported in comparative studies only, to inform other researchers on issues to consider when planning to use similar approaches.

A total of 118/140 (84%) of the included studies adopted a controlled design either using randomised (*n* = 104, 88%) [26–129] or non-randomised quasi-experimental approaches (*n* = 14, 12%) [130–143]. These were reviewed further and described as presented in Table 4 illustrating the type of controls used and how the study groups were allocated. A list of studies in each category is presented in S2 File.

**Table 4:** Description of research methodologies showing randomisation approaches used in the reviewed comparative studies (*N* = 118), the type of control used, and the number of studies in each category.

Interventions for Treatment and therapy HDs (*n* = 45)			
Study designs	Randomisation/group allocation	Control type	*n*
Randomised	Intervention: Control	Usual care	25
		Active/Alternative intervention	5
		Waitlist	6
	Intervention 1: Intervention 2: Control	Usual care	2
		Active/Alternative intervention	2
	Intervention: Control 1: Control 2	Active/Alternative intervention	1
Non-randomised	Intervention: Control	Historic	2
	Intervention: Control (1:3 ratio)	Historic	1
	Intervention: Intervention healthy population: Waitlist control healthy population	Healthy population Waitlist	1
**Self-management HDs (*n* = 45)**			
Randomised	Intervention: Control	Usual care	25
		Active/Alternative intervention	7
		Waitlist	1
	Intervention 1: Intervention 2: Control	Usual care	2
		Waitlist	1
Non-randomised	Intervention: Control	Usual care	1
		Historic	4
		Active/Alternative intervention	1
		Non-responders	1
	Intervention 1: 2 × Intervention 2: 2 × Control	Usual care	1
	Intervention: 2 × Control	Historic	1
**Preventive behavioural change HDs (*n* = 28)**			
Randomised	Intervention: Control	Usual care	8
		Active/Alternative intervention	13
		Waitlist	4
	Intervention 1: Intervention 2: Control	Usual care	1
		Waitlist	1
Non-randomised	Intervention: Control	Waitlist	1

#### Randomised controlled designs.

The majority of the reviewed RCTs used *usual care* (*n* = 63, 60%) as comparators. Other types of comparators comprised *active control/alternative interventions* (*n* = 28, 27%) and *waitlist/delayed* interventions (*n* = 13, 13%).

When grouping according to conditions, *usual care* was the most common comparator employed in controlled studies for all conditions, present in 36%, (*n* = 15) of cancer studies, 46% (*n* = 24) of diabetes mellitus studies, 55% (*n* = 21) cardiovascular studies and 75% (*n* = 6) of studies with multiple conditions. Additionally, waitlist/delayed treatment was the second most common comparator for cancer studies, employed in 24% (*n* = 10) studies whereas *active/alternative* intervention was the second comparator of choice for diabetes and cardiovascular studies. Historic controls were used little in studies across all conditions; 10% (*n* = 4) in cancer studies, 6% (*n* = 3) in diabetes mellitus studies, 8% (*n* = 3) in cardiovascular diseases, and none in studies with multiple conditions.

Of all the 63 RCT studies that employed usual care controls, 92% (*n* = 58) had equal (1:1) and random allocation of participants to two parallel groups, i.e., between intervention and control. Five of the sixty-threee RCTs were three-arm studies, with equal and random allocation of participants to two interventions and one usual care control group.

Of all the 28 RCTs which used active/alternative intervention control, 89% (*n* = 25) equally and randomly allocated participants to two parallel groups, i.e., to either intervention or an active/alternative intervention control group. The remaining three RCTs were parallel arm trials2014two of which compared two interventions against an active control, and one study compared an intervention against two active controls (Table 4).

Of the 13 RCTs which used waitlist/delayed intervention control, participants were equally and randomly allocated between the intervention or waitlist/delayed intervention in 11 studies. Two studies were three-arm trials comparing two interventions against one waitlist control (Table 4).

#### Non-randomised controlled designs.

For the non-randomised quasi-experimental studies, historic controls were the predominant comparators, used in more than half of the studies (8/14). Among these, six (Table 4) compared intervention groups with 1:1 matched historic controls, while two studies compared an intervention group with matched historic controls that were twice as large as the intervention groups (i.e., 1:2 ratio). One study used a second setting as the comparator group providing an alternative non-DH intervention (active control). Two studies had usual care as comparators. One study was conducted in a real-life clinical setting, and the groups were formed sequentially with a control group formed after the clinic stopped the DH intervention—thus patients were receiving usual care. In a second study, two interventions were compared to a usual care control group in a ratio of 1:2:2 (the first intervention group was smaller than the second intervention and the control groups). Two studies used control groups which had delayed access to an intervention. One of these studies used 1:1 allocation and both groups were drawn from an ill population, while in the second study, a healthy population group was used as a comparator and was further divided and randomly assigned to either receive an intervention or to a waitlist group making it a three-arm trial. The last study used an equal number of the intervention’s non-responders as a control group.

### Authors reported methodological challenges in comparative studies evaluating the effectiveness or efficacy of DHT interventions

We present the author-reported limitations in the context of methodological challenges found in the pilot and full studies included in the review.

### Author-reported limitations in the pilot trials

Fourteen of the one hundred eighteen (12%) comparative studies included in the review were either pilot or feasibility studies. Eight author-reported limitations were reported, and these were summarised into four themes including: (1) issues pertaining *length of the study*, (2) limitations in *recruitment* ,(3) limitations in eva*luation and measurement techniques*, and (4) the presence of *Confounding variables*. Table 5 presents a list of the limitations reported grouped by theme and their frequency of reporting.

**Table 5:** Author-reported limitations in pilot studies summarised into themes and the frequency of reporting each limitation and themes.

Limitations theme	*n* (%) theme was reported (*N* = 29)	List of limitations	*n* (%) of studies reported (*N* = 14)
Recruitment	12 (41)	Inadequate sample size due to under-recruitment	6 (43)
		Inadequate sample due to high attrition	1 (7)
		Bias in selection and recruitment of participants	5 (36)
Length of the study	3 (10)	Short duration of the study/follow-up	3 (21)
Evaluation/measurement techniques	9 (31)	Unreliability/validity of measurement tools	5 (36)
		Issues with intervention compliance and missing data	4 (29)
Confounding variables	5 (17)	Inability to control study environment or group exposure to intervention (contamination)	3 (21)
		Inherent systemic difference between study groups	2 (14)

S4 File presents a list of pilot/feasibility studies reporting each limitation grouped according to the aim of DHT intervention.

### Author-reported limitations in full comparative studies included in the review

We reviewed the limitations reported in 104 full studies that adopted a comparative approach in their designs and 13 limitations were found. These were assigned to four themes similar to those in pilot studies namely: (1) *recruitment*, (2) *study length,* (3) *confounding variables,* (4) *evaluation and/or measurement techniques*. Table 6 shows the limitations grouped into the four themes and their frequency of reporting. S5 File presents a list of full comparative studies reporting the limitations grouped according to the condition investigated and the aim of DHT intervention.

**Table 6:** Author reported limitations in full comparative studies summarised into themes and the frequency of reporting each limitation and themes.

Theme	*n* (%) theme was reported (*N* = 209)	Limitations	*n* (%) of studies reported, (*N* = 104)
Recruitment	78 (37)	Small sample due to under-recruitment	34 (33)
		Small sample due to high attrition rate	4 (4)
		Bias in selection and recruitment of participants	40 (38)
Length of the study	24 (11)	Short duration of the study	24 (23)
Evaluation/measurement techniques	57 (27)	Unreliability/validity of measurement tools	23 (22)
		Unstandardised study procedures/changes in protocol/technical faults	6 (6)
		Low adherence to the intervention	17 (16)
		Incomplete or missing data/low adherence to protocol	4 (4)
Confounding variables	50 (24)	Multi-component intervention – difficult evaluating individual impact	8 (8)
		Inability to control study environment or group exposure to intervention (contamination)	13 (13)
		Expert/researcher influence on the outcome	14 (13)
		Inherent systemic differences between study groups	3 (3)
		Confounders not accounted for in evaluation	19 (18)

**Fig 2:**
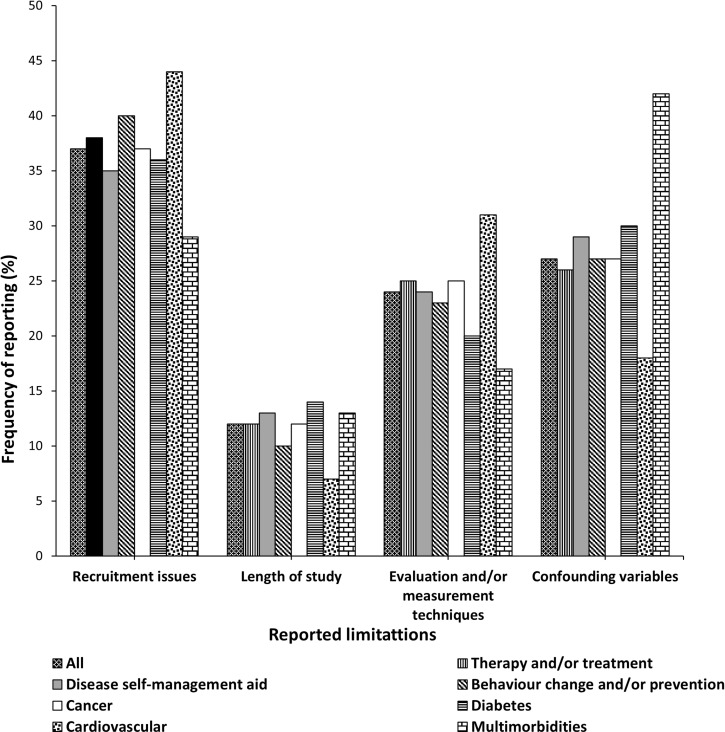
Themes of author-reported limitations and the frequency of reporting, grouped according to the condition investigated and aim of DH intervention.

Issues in *recruitment* were the most commonly reported limitation (37%). This was followed by *confounding variables*, limitations in *evaluation/measurement techniques* used, and the *length of the study* (Table 6). The same trend was observed when the data was grouped according to the condition investigated and the aim of the DHT intervention with few exceptions. The highest proportion of cardiovascular studies reported limitations in *recruitment* and *evaluation/measurement techniques* whereas those reported *confounding variables* and *length of the study* limitations were lower than the proportions for the studies overall. Another difference was seen in studies involving multimorbidity whereby the proportion that reported the presence of *confounding variables* was above the average whereas those reporting limitations in *recruitment* and *evaluation and/or measurement techniques* were below the average for the studies overall (Fig 2).

#### Limitations in recruitment.

**Fig 3:**
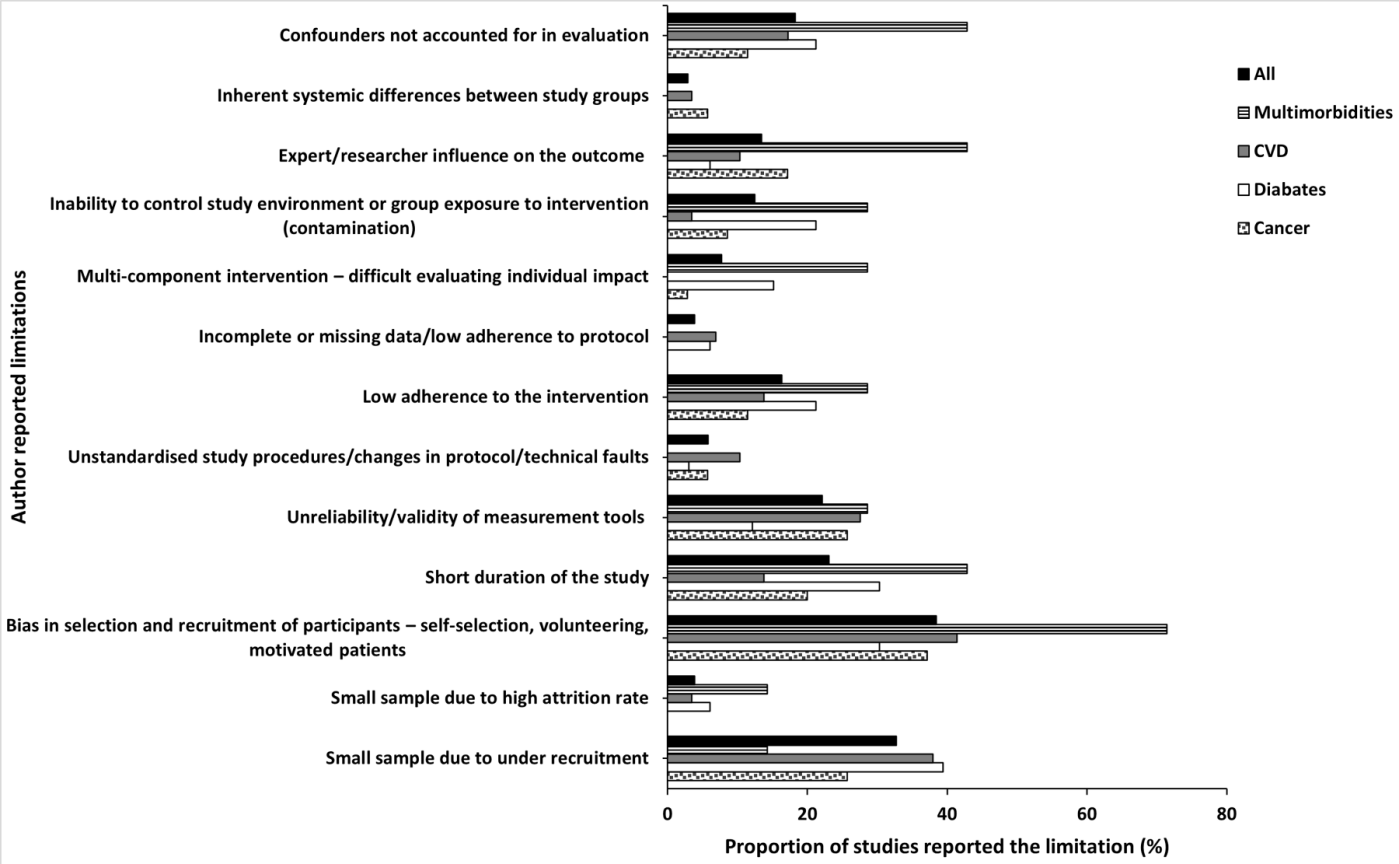
Author reported limitations and proportion of studies reported each limitation presented according to condition investigated.

**Fig 4:**
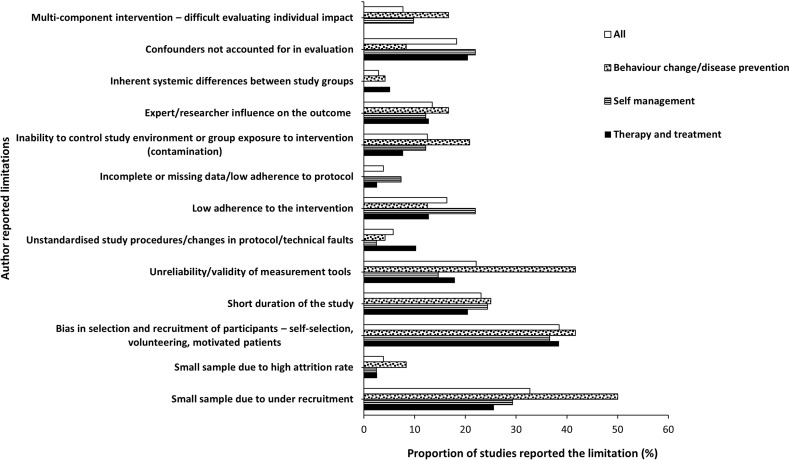
Author reported limitations and proportion of studies reported each limitation presented according to condition investigated.

The reported limitations in *recruitment* include *bias in selection and recruitmen*t of participants, which appeared in just over a third (38%) of the reviewed full comparative studies. Other limitations were *small sample size due to under-recruitment* (33%, *n* = 34) and *high attrition rate* (4%, *n* = 4). When grouped according to the conditions investigated, compared to the overall sample, a greater proportion of studies that recruited participants with multimorbid conditions reported *bias in selection and recruitment* (71%, *n* = 5). The proportion of diabetes (39%, *n* = 13) and cardiovascular (38%, *n* =11) studies that reported *small sample size due to under-recruitment* was also slightly higher than the overall percentage of 33% (Fig 3). When grouped according to the aim of DHT, studies of behaviour change and/or disease prevention interventions we identified a greater proportion reporting all three limitations in recruitment (Fig 4).

#### Presence of confounding variables.

Confounding variables reported include the presence of *expert/researcher influence* (13%, *n*= 14), *uncontrollable environmental factors* that influenced the findings (13%, *n* = 13), *inherent systemic differences between study groups* (3%, *n* = 3), and *multi-component intervention* (8%, *n* = 8). Eighteen percent (*n* = 19) of the studies acknowledged that their methodologies *did not account for confounding variables*. When grouped according to the condition investigated, studies with multimorbidity reported a greater proportion of all four individual confounding limitations when compared to the proportion for the overall sample (Fig 3). When grouped according to the aim of DHT intervention, the proportion of behaviour change and/or disease prevention intervention studies that reported the presence of *multi-component intervention*, *inability to control study environment (contamination)*, and *expert/researcher influence* were higher than that reported by the studies overall. Also, the proportion of studies of behaviour change and/or disease prevention intervention and those of disease self-management that did not account for *confounding variables in their evaluation* were greater than those in the overall sample (Fig 4)

#### Limitations in evaluation and/or measurement techniques.

The reported limitations in evaluation and/or measurement techniques include the use of *unvalidated/unreliability measurement techniques*, reported by nearly a quarter of the studies (22%, *n* = 23); *unstandardised study procedure or change in protocol or technical faults* during the study (6%, *n* = 6); low *adherence to intervention* (16%, *n* = 17) and *incomplete/missing data* (4%, *n* = 4). When grouped according to the conditions investigated, the proportion of studies that reported *low adherence to the intervention* was higher in multimorbidity studies compared to the proportion of the studies overall. All conditions in a similar proportion (above the average) reported being limited by *unreliable/unvalidated measurement tools* except diabetes studies which reported in lower proportion than average (Fig 3). When grouped according to the aim of the DHT intervention, a greater proportion of *incomplete/missing data and low adherence to interventions* was reported for self-management intervention studies. *Unreliability/validity of measurement tools* was most frequently reported for behaviour change/disease prevention interventions whereas a greater proportion of *unstandardised study procedure/changes in protocol/technical faults* were reported for therapy/treatment intervention studies than in the overall sample (Fig 4).

#### Length of the study.

Nearly a quarter of the studies (23%, *n* = 24) reported that the interventions or their studies were *short and/or had inadequate follow-up periods*. When grouped according to the condition investigated, a greater proportion of multimorbidity and diabetes studies reported this limitation than the studies overall (Fig 3). When grouped according to the aim of the DH intervention, the proportion of disease self-management and Behaviour change/disease prevention studies that reported being of short duration was slightly above that of the studies overall (Fig 4).

## Discussion

### Methodologies used in DHT evaluation studies

Our review found that RCTs are the primary methodological choice for most researchers evaluating DHT interventions, independent of the health condition investigated or the purpose of the DHT intervention, i.e., *providing treatment/therapy, aiding self-management*, or *preventive/behavioural change.* This is consistent with the findings of recent reviews [17,18] indicating that, despite its perceived limitations, the RCT design is still the preferred approach for evaluating DHT interventions. The majority of studies used the standard two-arm parallel design in which participants are randomly allocated between the intervention or control group. Again, these approaches to evaluating DHT interventions are perhaps surprising given that in most cases there may be multiple elements influencing their effectiveness.

Multi-arm trials have been suggested as an alternative design, offering the possibility of conducting multiple comparisons simultaneously [144]. In the present review, there were ten RCTs with more than two trial arms. Those with multiple arms compared either two interventions with one control or one intervention compared with two control groups. Nevertheless, it is important to note that where multiple comparisons are made simultaneously within the same study (e.g., subgroup comparisons, comparisons across multiple treatment arms, analysis of multiple outcomes, and multiple analyses of the same outcome at different times), the probability of rejecting at least one null hypothesis given that all nulls are true increases (type I error), misleading the findings (false-positive findings) [145]. In this case of multiplicity, adjustments need to be made in terms of the design and statistical analysis plan. For example, a single outcome/comparison needs to be identified as the primary and treat the remaining outcomes/comparisons as extensively discussed in studies by Li and colleagues and Odutayo and colleagues [144,146]. These did not feature in the studies with multiple controls included in the present review.

Some studies used cluster randomisation by grouping the individuals into clusters (groups) and then randomising these groups to different study arms [32]. Cluster randomisation is increasingly being adopted when evaluating complex interventions, particularly those with multiple individual elements that are likely to interact or influence each other, an issue that is significant in most DHT interventions [147]. However, cluster randomised trials tend to be complex to design, deliver and even interpret, compared to individual RCTs due to the interplay between the similarities and differences within and between clusters respectively [148,149]. Therefore, care needs to be taken when deciding on the sample size to ensure studies are sufficiently powered, avoid bias in selection and recruitment, and consider suitable data analysis approaches and reporting [150,151].

Our review also highlighted the small number of non-comparative studies, such as pre-post single-arm follow-up, cross-sectional observation, mixed methods, and retrospective studies. Temporal variability is thought to be more problematic in these types of designs as, without a control group, there is no way of knowing what the outcome would have been without an intervention [152]. To account for the lack of control, multiple measures of the outcome of interest at baseline and after intervention are suggested [153].

### Type of control/comparator group

The present review also illustrated that *usual/standard care* is the preferred comparator for most researchers. While it is recognised that a placebo control is an ideal comparator when designing high-quality RCTs, there have been ethical debates regarding the use of a placebo in health intervention studies as it would mean denying some of the study participants care/treatment and possibly exposing them to further harm or risk [154]. Also, because of the nature of the DH intervention, i.e., inability to mask the intervention, it is questionable whether a placebo control could be feasibly employed and/or be of benefit. Active controls have increasingly been adopted in clinical intervention studies to address the ethical concerns about placebo control. In the present review, 21.4% of studies compare the intervention of interest with an alternative intervention/active control. Nevertheless, the use of active control is not without its own limitations. Researchers have highlighted the lack of sensitivity in such trials which measure the relative efficacy of the intervention of interest relative to the alternative, unlike in placebo-controlled designs which enable assessment of the absolute efficacy of the intervention [155].

We also found waiting list/delayed treatment controls were used, but this was not common (in 13 of the 104 RCTs). The use of waitlist controls is ethically advantageous since it allows the provision of the same care/treatment to both study arms. However, it is argued that such designs run the risk of overestimating the intervention effect as unexpectedly lower levels of the variable of interest tend to be observed in waitlist control groups. This is particularly true in studies where participants are aware of their group allocation as they are likely to consciously not show/inform any changes until they start the intervention [156]. Considering the nature of DHT interventions (i.e., difficult to mask), it is also often likely that participants in the control group will be exposed to or access the intervention either intentionally or unintentionally, contaminating the study and creating biased results. This concern was acknowledged by some of the reviewed studies [40,94,95,120]. Finally, the use of a waitlist control group precludes assessment of the long-term effect of the intervention.

Fourteen studies were controlled but not randomised, with eight of them using historic groups/data as a comparator. Historic controls have commonly been used in situations where there are ethical concerns or where it is impractical to have a concurrent control group [157]. However, the use of historic controls can lead to selection bias or failure to account for systematic differences between the groups or data and conditions/standards of the studies [158,159], importantly in the fast-paced technology era as acknowledged in two studies included in our review [134,140]. Six of eight historically controlled trials included in the present review used matched historic control groups whereas the remaining two studies used historic comparison groups that were twice as large as the intervention groups. The use of non-responders and healthy individuals as control groups was also noted, confounding the results due to possible inherent differences between groups.

### Author-reported limitations in comparative studies

#### Limitations in recruitment.

Nearly half of the pilot studies and more than half of full trials reported limitations in *recruitment*, particularly *inadequate sample size due to under-recruitment* and *bias in selection and recruiting* participants. While it is common for pilot or feasibility studies to have a small sample size since they are usually conducted to inform the design of future full trials rather than to measure effectiveness directly themselves, in our review, over a quarter of the full trials also reported under *recruitment* and over one-third reported *bias in selection and recruitment* compromising the internal and external validity of the results. This was particularly seen in a higher proportion of studies evaluating behaviour change/disease prevention studies and those studying multimorbidity respectively. Under recruitment in online studies has been reported elsewhere and the recruitment strategy employed seems to play a role in recruitment outcomes [160,161]. Online recruitment methods for example through social media are increasingly used in intervention studies including those in the present review as they have the potential for a wide reach. However, this does not always translate to a positive response. Online messages can be perceived as fraudulent information and unsafe and people can be reluctant to respond or ignore the adverts [162]. Moreover, although online recruitment might be a reasonable recruitment strategy for interventions delivered and accessed through technology, it carries the risk of creating a sample that is not representative as participants recruited online are subject to greater self-selection and may disproportionately comprise people from a particular socio-economic group, technology savvy or those who are health conscious or motivated to seek health solutions [163,164]. Recruitment strategies must consider individual differences and tailor the recruitment strategy depending on the targeted population [165]. Where online recruitment methods are used, it is worth using trusted and/or verified sources for example institutional platforms, and ensure the data protection strategy is clear and communicated [166]. One could also consider using offline approaches (such as word of mouth through community networks or letters) in parallel to online recruitment [162]. Budgeting for and promising provision of technology and/or technology skills and ongoing technical support throughout the intervention might help to increase confidence of research volunteers and widen participation and inclusion [166].

#### Length of study.

Some of the reviewed studies had short intervention periods, in some cases as short as 4–8 weeks. This was the case for both pilot and full trials, while this might not be a concern for pilot trials, it is more problematic in full trials aiming to demonstrate impact or whether an effect dissipates over time, particularly for lifestyle or behavioural change interventions, which require time to work. In our review, a quarter of the studies which evaluated DHT for behavioural change or disease prevention and more than one-third of multimorbidity studies reported being of short duration. Longer follow-up of at least 1 year would generally be expected for behavioural change or risk prevention interventions to enable impact as recommended by NICE guidance on individual approaches to behaviour change [167]. However, designing long DHT interventions might be challenging due to the fast-paced nature of technology. Nevertheless, the level of engagement or exposure to interventions is equally important and critical for the effectiveness of DHT, and this extends beyond the study duration encompassing the amount, frequency, depth, and length of usage of the intervention [168]. Thus, engagement is an important measure in DHT evaluation studies despite the challenges to accurately capture such data due to its manifaced nature [169,170]. In the present review, although user engagement was not among the reported limitations, it is possible that this was not well evaluated in the studies limiting the understanding of the interventions’ impact.

#### Evaluation and measurement techniques.

A good proportion of studies reported being limited by the use of measurement tools/scales that are unvalidated and unreliable or that they collected participants’ self-reporting data which can be unreliable. This is particularly the case for studies on behaviour change/disease prevention and those with multimorbidity. For example, despite the use of validated standard outcome measures such as distress (e.g., Perceived Stress Scale [171]), anxiety (e.g., Generalized Anxiety Disorder 7-item Scale (GAD-7) [172]) and depression (e.g., Patient Health Questionnaire-9 (PHQ-9) [173]), health-related quality of life (e.g., Assessment of Quality of Life 8-item Questionnaire [174]), participants self-report is thought to be subject to bias, particularly when participants are aware of the intervention they are receiving and the expected outcome. Participants’ technology skills and awareness of their condition (e.g., those on long-term conditions) may contribute to bias in reporting. Additionally, other outcome measures like dietary intake and physical activities rarely used standardised scales [175]. Training of participants to accurately record and report data, employing blinding techniques and incorporating objective measures where possible capturing data directly by the digital devices might help reduce systemic error in reporting data.

#### Confounding variables.

Nearly a quarter of full comparative trials reported being confounded by different factors such as the inability to control the study environment and the influence of a researcher or experts involved in the delivery of the intervention to the outcome of the intervention. In particular, this was highly reported in studies evaluating behaviour change/disease prevention intervention, perhaps because varied individual environmental factors may unwittingly influence their behaviour and findings. It is also difficult to blind behavioural interventions [176]. Authors reported contamination where the participants from control groups gained access to the intervention either intentionally or unintentionally [177]. This is also common for study designs using waitlist and/or standard care as usual controls and not blinded to intervention allocation [178]. In the present review, of the 13 studies that reported contamination as a limitation, 9 used usual care as the control condition and 4 employed a waitlist control. Psychotherapy research has demonstrated small effect sizes in studies with waitlist control groups [179] suggesting that caution needs to be taken when using this type of control to account for the waiting effect. Participants’ awareness of their allocation might also influence how they respond within a trial and so potentially leading to biased responses [180]. Few considerations have been suggested to mitigate the highlighted limitations such as the adoption of pragmatic randomised control trials by employing appropriate alternative intervention choices as a control group rather than no-treatment or usual care [181]. The use of cluster randomisation and mantling designs like MOST and the SMART which allows variations of intervention content delivered to participants helps to evaluate the isolation effects of different components of interventions and different times [16].

### Strengths and limitations of the study

The strength of our scoping review is boosted by the fact that we conducted an extensive search and reviewed a large body of literature. Narrowing our review focus to three long-term conditions of global relevance in terms of disease prevalence and impact, and with high adoption of DHT has enabled us to synthesise the literature rigorously and produce evidence that is more relevant and with wide impact. Our review is limited in the sense that, due to the fast-paced proliferation of DHT and the associated evaluation studies, the evidence presented might not be the latest at the time of publication. Nevertheless, we believe that the conclusion and recommendations made will remain relevant and applicable to research contexts similar to those included in the present review.

## Conclusion

Our review suggests that the standard, two-arms parallel RCT design remains the preferred methodological approach with the *usual/standard care* group commonly used as the control arm. The reported methodological limitations including selection and recruitment bias suggest it is worth considering recruitment modalities that are inclusive using online and offline credible channels. Additionally, RCTs can be seen as restrictive and time-consuming, unable to react to the fast pace of development of technology-based solutions; particularly those involving artificial intelligence or machine learning [182]. Adoption of inclusive approaches to recruitments and emerging pragmatic approaches to RCTs including mantling designs such as the MOST and SMART methods holds considerable promise in addressing the fast-paced, multi-component and group contamination problem resulting from the unconcealable nature of DHT interventions.

## Supporting information

S1 File Search strategy. (DOCX)

S2 File List of studies included. (DOCX)

S3 File Data extraction form. (DOCX)

S4 File Limitations pilot studies. (DOCX)

S5 File Limitations full studies. (DOCX)

S6 File PRISMA-ScR checklist. (DOCX)
