# Supplementary material for: Methodological approaches and author-reported limitations in evaluation studies of digital health technologies (DHT): A scoping review of DHT interventions for cancer, diabetes mellitus, and cardiovascular diseases
Source: PLOS Digit Health. 2025 Apr 24;4(4):e0000806. doi: 10.1371/journal.pdig.0000806 (PMC12021190; doi:10.1371/journal.pdig.0000806)
Supplement: S1 File — (DOCX) [file pdig.0000806.s001.docx]

**Appendix A: Search strategy used in the literature review (first search conducted between March – June 2020) and number of records results**

**Common conditions applied**: Publication date: 2016- 2020; Publication type: journal articles (peer reviewed); English language; Human studies; Population groups: Adults patients; Abstract available

| Database | Digital health | Health care | Database specific filters | Results |
| --- | --- | --- | --- | --- |
| Medline | Telemedicine OR telerehabilitation OR “remote consultation” OR “internet-based intervention” OR “mobile application” OR smartphone OR “cell phone” OR “Therapy, computer assisted” | “delivery of health care” OR “health” OR “health promotion” OR “disease management” OR “treatment outcome” OR therapeutics OR “rehabilitation OR “patient care” OR nursing | Clinical trial, adult (19+ years), | 224 |
| Embase Elsevier | Telehealth OR telemedicine OR “mobile health” OR “mobile application” OR “web-based intervention” OR “online pharmacy” OR “e-counselling” OR “online monitoring” OR “computer assisted therapy” OR “mobile phone” OR smartphone | Health OR “Health care” OR “health care access” OR “home care” OR “health service” OR “health care delivery” OR “health care practice” OR “nursing, treatment outcome” OR “outpatient care” OR wellbeing OR “health promotion” OR therapy OR “disease management” OR “quality of life” OR rehabilitation | intervention study | 279 |
| PsycINFO | Telemedicine OR “digital interventions” OR “digital technology” OR website OR “online therapy” OR “assistive technology” OR “computer assisted therapy” OR “mobile health” OR “smart phones” OR “mobile applications” | "Health Care Delivery" OR "Clinical Practice" OR "Health Care Access" OR "Health Care Utilization" OR "Managed Care" OR "Quality of Care" OR "rehabilitation” OR “disease management” OR “health promotion” | Adults (18+ years) | 1699 |
| CINAHL | Technology OR smartphone OR “cellular phones” OR “mobile application” OR computers OR telemedicine OR telerehabilitation OR telehealth OR telepsychiatry OR “wearable sensors” | Health OR “Outcome (health care)” OR “healthcare delivery” OR “nursing interventions” OR “health promotion” OR “disease management” OR “quality of life” OR well-being OR wellness |  | 4673 |
| Web of Science | Telemedicine OR  “Mobile Health” OR  MHealth OR Telehealth OR eHealth OR “mobile applications” OR “internet-based intervention” OR digital OR smartphone OR “cell phone” OR “computer assisted therapy” | Health OR “Health behaviour” OR “Health promotion” OR “delivery of health care” OR “health services” OR “disease management” OR therapy/therapeutic OR rehabilitation OR “patient care” OR “quality of life” OR “treatment outcome” OR nursing | Document type: article; excluding: Proceedings paper OR book chapter OR data paper OR early access | 16134 |

**Appendix B: The EBSCOhost search string used for the second search (update search conducted in March 2022)**

| **Database** | **Search query** | **Limiters** | **Results** |
| --- | --- | --- | --- |
| EBSCOhost search (Medline + CINAHL + PsycINFO) | SU (Telemedicine OR telerehabilitation OR “remote consultation” OR “internet-based intervention” OR “mobile application” OR smartphone OR “cell phone” OR “Therapy, computer assisted”) AND SU (“delivery of health care” OR “health” OR “health promotion” OR “disease management” OR “treatment outcome” OR therapeutics OR “rehabilitation OR “patient care” OR nursing ) AND SU ( diabetes OR cancer OR hypertension OR cardiovascular ) | Published Date: 20200501 - 20220331.  English Language: Peer Reviewed; Human; Age Related: All Adult: 19+ years; Research Article.  **Expanders**  Apply related words; Apply equivalent subjects  **Search modes**  Boolean/Phrase | 464 |
