# Supplementary material for: Methodological approaches and author-reported limitations in evaluation studies of digital health technologies (DHT): A scoping review of DHT interventions for cancer, diabetes mellitus, and cardiovascular diseases
Source: PLOS Digit Health. 2025 Apr 24;4(4):e0000806. doi: 10.1371/journal.pdig.0000806 (PMC12021190; doi:10.1371/journal.pdig.0000806)
Supplement: S2 File — (DOCX) [file pdig.0000806.s002.docx]

| **Condition** | **Author, year** | **DH Intervention** | **Aim of DH** | **Study design** | **Comparator** | **Duration of study** | **Aim of study** | **Reported methodological issue/limitations.** |
| --- | --- | --- | --- | --- | --- | --- | --- | --- |
| **DH aiming at providing treatment and therapy (n = 45)** | | | | | | | | |
| Cancer  (n = 20) | [Abrahams et al., 2017](https://pubmed.ncbi.nlm.nih.gov/28621820/) | Internet based-Cognitive Behavioural therapy with web-based modules, electronic consultation with behavioural therapist | Improve fatigue, functional capacity and psychological distress | Randomised controlled trial with two parallel groups | usual care | 24 weeks (6 months) | To examine the efficacy of Internet-based cognitive behavioural therapy (ICBT) for severe fatigue in survivors of breast cancer | No blinding; Ethical concerns of denying intervention to a control; Short duration of intervention – long-term impact unknown. Controlled follow-up assessments for control group could not be incorporated into the design |
|  | [Shah et al., 2021](https://pubmed.ncbi.nlm.nih.gov/32567391/) | Post operative phone calls and virtual wound checks (photo or video conference) with physician or nurse practitioner | Reducing emergency room (ER) visits and readmissions. | Controlled, non-randomised | Historic control | 56 weeks (12 months) | Evaluate the impact of a patient phone calls and virtual wound checks  on reducing emergency room (ER) visits and readmissions | time of day when phone calls were made was not standardized due to the unpredictable nature of the surgeon’s and nurse’s daily schedule which may limit contacts and response.  Patient’s understanding of their own condition and family support system might impact outcome |
|  | [Ariza-Garcia et al., 2019](https://www.ncbi.nlm.nih.gov/pmc/articles/PMC6685131/#:~:text=The%20results%20of%20this%20RCT%20show%20that%20a,patients%20with%20breast%20cancer%20who%20are%20undergoing%20chemotherapy.) | Low-intensity Web-based therapeutic exercise program | Improving the functional capacity, strength, anthropometric parameters, and body composition of patients with breast cancer | Randomized controlled trial with two parallel groups | usual care | 8 weeks | To evaluate the effectiveness of intervention on functional capacity, strength, anthropometric parameters, and body composition | Unreliability of treadmill to measure walked distance – risk of overestimation; Short duration of study – long term impact unknown |
|  | [Bellens et al., 2020](https://pubmed.ncbi.nlm.nih.gov/32554133/) | Web-based cognitive rehabilitation/training through video game | Improving cognitive functioning | Randomized controlled trial with cross-over control | Waitlist | 24 weeks (6 months) | Investigate whether web-based the intervention is a feasible approach to treat cognitive decline in combination with our standard of care | Inadequate sample size (Pilot study); Recruitment bias – participants recruited based on their personal perception of suffering from cognitive dysfunction. Limits generalizability to general population of breast cancer survivors; Low compliance to the intervention; Limitation of subjective measures of cognitive functioning – better assessment with standardised and objective measures |
|  | [Compen et al., 2018](https://pubmed.ncbi.nlm.nih.gov/29953304/) | Internet-based mindfulness-based cognitive therapy (eMBCT) with weekly asynchronous written interaction with a therapist over e-mail | Reduce psychological distress among patients with cancer; Improve Symptoms; Physical functioning | Randomised controlled trial, with three parallel groups (2 interventions + 1 control) | Usual care | 8 weeks | To investigate whether F-to-F and Internet web-based MBCT intervention is superior to the usual care in reducing psychological distress in a sample of distressed patients with cancer | Two interventions but study not powered to directly compare them or establish noninferiority; Bias in recruitment – middle aged; Bias in groups allocation by preference; Short duration hence long-term effect unknown; Multi-component intervention – difficult assessing active element |
|  | [Dos Santos et al., 2020](https://www.ncbi.nlm.nih.gov/pmc/articles/PMC7756299/) | Computer‐assisted cognitive rehabilitation (CR) program | To improve cognitive function | Randomized controlled trial with three parallel groups (1 intervention + 2 controls) | Active control | 12 weeks | To evaluate the impact of computer assisted intervention on cognition, QOL, anxiety, and depression among cancer patients treated with chemotherapy. | Short duration – long term impact unknown; Recruitment bias – predominantly breast cancer with previous hormone therapy (controversial effect on cognitive functioning). Presence of neuropsychologist in EG might impact findings. |
|  | [Hauffman et al., 2020](https://www.ncbi.nlm.nih.gov/pmc/articles/PMC7821133/) | Internet‐based stepped psychosocial health care (iCAN‐DO) | To improve psychosocial behaviour and depression symptoms | Randomized controlled trial with two parallel groups - Multicentre | Usual care | 40 weeks (10 months) | To evaluate the effects of intervention on anxiety, depression, posttraumatic stress, and health‐related quality of life (HRQoL) in individuals with cancer and self‐reported anxiety and/or depression symptoms | Bias recruitment – predominantly young, with more favourable prognosis – limits generalizability of results; High attrition /lost to follow up |
|  | [Holtdirk et al., 2021](https://www.ncbi.nlm.nih.gov/pmc/articles/PMC8104369/) | CBT-based, holistic Internet-based intervention -Optimune | Improving symptoms of depression, anxiety, and fatigue and breast cancer survivors | Randomised controlled trial with two parallel groups | Usual care | 56 weeks (12 months) | To test effects of Optimune on quality of life (QoL) and dietary habits and physical exercise | A novel unvalidated questionnaire used for dietary assessment; Unreliability of self reported measures; There was limited data collected on disease info limiting comparison statistics; Recruitment/selection bias – highly educated, motivated and with internet engagement |
|  | [Huang et al., 2021](https://pubmed.ncbi.nlm.nih.gov/33771645/) | web-based health education program for symptoms management | Improve Quality of life and Symptom Distress | Randomised controlled trial with two parallel groups | usual care | 12 weeks | To evaluate the effects of a web-based health education program on global quality of life, quality of life-related functional dimensions, and symptom distress of initially diagnosed advanced non-small cell lung cancer patients. | Important confounding factor not considered – tech savvy; Lack of user instructions and recorded browser history; Finding cannot generalised to all advanced NSCLC only those treated with chemo |
|  | [Hummel  et al., 2017](https://pubmed.ncbi.nlm.nih.gov/28240966/) | Internet-based Cognitive behaviour therapy for sexual health | Improve sexual function; Reduce sexual distress | Randomised controlled trial, with two parallel groups | Waitlist/delayed intervention | 36 weeks (9 months) | To investigate the efficacy of Internet-based CBT in improving sexual functioning in BC survivors with diagnosis of a sexual dysfunction | Low completion rate, 62% of participants; Budget constraint limited follow-up and it was not possible to conduct exit interview to establish changes in diagnosis of sexual disfunction |
|  | [Kim et al., 2020](https://pubmed.ncbi.nlm.nih.gov/32779223/) | e-health interventions for positive psychosocial changes in breast cancer patients | To support illness management and treatments | Randomised controlled trial with three parallel groups (2 interventions + 1 control) | Active control/Alternative intervention | 24 weeks (6 months) | To explore how using different e-health interventions facilitates positive psychosocial changes and how these changes reduce cancer concerns and improve quality of life in breast cancer patients over time | Findings may only be generalizable to breast cancer patient populations that are open to using an e-health intervention; Recruitment/selection bias – education level of participants was higher than the national average; Reliability score of information overload was low |
|  | [Lleras de Frutos et al., 2020](https://pubmed.ncbi.nlm.nih.gov/32618395/) | Online positive psychotherapy intervention for cancer survivors | To promote positive emotions, strengths, and personal meanings, to reduce emotional distress an post-traumatic stress symptoms | Pragmatic Randomized controlled trial –parallel comparing two modalities of therapy | Active control | 12 weeks (3 months) | To examine the impact of an online positive psychotherapy in cancer (OPPC) group, held via videoconference compared to face-to-face | The respect for patient treatment preferences may have partly biased the results – no complete randomisation; Lack of nontreatment control group |
|  | [Maguire et al., 2021](https://www.ncbi.nlm.nih.gov/pmc/articles/PMC8293749/#:~:text=eSMART%20aimed%20to%20provide%20definitive%20high%20quality%20evidence,cancer%2C%20colorectal%20cancer%2C%20Hodgkin%E2%80%99s%20disease%2C%20or%20non-Hodgkin%E2%80%99s%20lymphoma.) | Advanced Symptom Management System (ASyMS) for real-time remote monitoring of adjuvant chemotherapy related side effects | To support real-time monitoring and management of chemotherapy toxicity | Randomised controlled trial with two parallel groups, multicentre | Usual care | 3 yrs | To evaluate effects of remote monitoring of adjuvant chemotherapy related side effects via the Advanced Symptom Management System (ASyMS) on symptom burden, quality of life, supportive care needs, anxiety, self-efficacy, and work limitations. | Three quarters of participants had breast cancer and were female – limit generalizability; Technical challenges across all sites owing to the connectivity of ASyMS SIM cards, meaning that patients using ASyMS reverted to standard care for approximately two weeks to ensure patient safety while this technical problem was resolved. |
|  | [Mihuta et al, 2018](https://pubmed.ncbi.nlm.nih.gov/29266524/) | Web-based cognitive rehabilitation program based on principles from cognitive- behavioural therapy (eReCog) – training modules | Improving cognitive function, distress, and quality of life of cancer survivor | Randomised controlled trial, with two parallel groups | Waitlist/Delayed intervention | 4 weeks | To examine the efficacy of a web- based cognitive rehabilitation | Recruitment bias – predominantly breast cancer, limiting generalizability of results to other cancer types; Lack of control for cancer group; Appropriateness of WebNeuro to measure cognitive functioning in adult cancer is questionable; Statistical significance and trends should be interpreted with caution as multiple comparisons were conducted |
|  | [Mihuta et al., 2018](https://onlinelibrary.wiley.com/doi/10.1111/ecc.12805) | Web-based cognitive rehabilitation therapy program (eReCog) -Interactive psychoeducation for cognitive function | Improving cognitive function, distress, and quality of life of cancer survivor | Non randomised, controlled trial. 1 Cancer intervention group + 2 Non cancer control groups (randomly assigned to parallel + Waitlist control) (Pilot study) | Wait list/delayed intervention | 12 weeks | To evaluate the efficacy of the web based Cognitive rehabilitation adapted from original face-to-face version | Relatively small sample size; No cancer group control - comparison was made with non-cancerous group. |
|  | [Urech et al., 2018](https://www.ncbi.nlm.nih.gov/pmc/articles/PMC5844668/) | Web-based stress management intervention, STREAM - cognitive behavioural– and mindfulness-based stress reduction techniques | Psychological support for stress management | Randomised controlled trial with two parallel groups | Wait-list/Delayed intervention | 16 weeks | To assess the efficacy of the intervention on stress management for newly diagnosed patients with cancer receiving first-line treatment | Possible variation on assessment between groups; Limited follow up of measures |
|  | [Watson et al., 2017](https://onlinelibrary.wiley.com/doi/10.1002/pon.4338) | Telephone-Delivered Cognitive Behavioural Therapy for cancer patients with high psychological needs | Reduce anxiety, depression and worries | Randomised controlled trial with two parallel groups | Usual care | 12 weeks | To evaluate the impact of Telephone Vs Treatment as Usual therapy on mental health and coping in cancer patients | Small sample due to under-recruitment; Recruitment bias - Unstandardised screening methods of participants, used clinical judgement of psychological need |
|  | [Wen et al., 2020](https://www.ncbi.nlm.nih.gov/pmc/articles/PMC7668526/) | Multimedia Education in colonoscopy delivered via Smartphone | To support bowel preparation and care for patients undergoing colonoscopy | Randomised controlled trial with two parallel groups | Usual care | 16 weeks (4 months) | To examine the effectiveness of smartphone education on bowel preparation, embarrassment, and satisfaction with care among patients receiving a colonoscopy. | Unable to monitor and document the times and frequency of watching the video hence could not evaluate dose-response effect; Did not account for/control participants previous experience with colonoscopy; Single centre study – possible bias limiting generalizability |
|  | [Zachariae et al., 2018](https://www.ncbi.nlm.nih.gov/pmc/articles/PMC6093474/) | Internet-delivered CBT-I (iCBT-I) | Management of insomnia | Randomised controlled trial with two parallel groups | Waitlist/ delayed intervention group | 15 weeks | To test the efficacy of iCBT-I in breast cancer survivors with clinically significant sleep disturbance | Recruitment bias – young and pre-menopausal; Unreliability of self-reported sleep data; Short follow-up period; Lack of parallel group comparison |
|  | [Vos et al., 2021](https://pubmed.ncbi.nlm.nih.gov/34224671/) | Cancer survivor care through eHealth app (Oncokompas) | To improve quality of life and survival of cancer patients’ post-surgery | Randomised controlled trial, pragmatic two-by-two factorial (  GP; GP +App; Surgeon; Surgeon + App  ), open label | Active control | 12 months | To assess the impact of eHealth app on patients’ quality of life | Blinding was not possible.  Crossover between intervention groups (inevitable due to healthcare system, open label)  Validity of QoL assessment tools questionable |
|  | [Akinci et al., 2018](https://pubmed.ncbi.nlm.nih.gov/29417832/#:~:text=The%20effects%20of%20Internet-based%20exercise%20compared%20with%20supervised,life%2C%20and%20both%20are%20better%20than%20simply%20counselling.) | Online aerobic and resistance training program with reporting system | To improve physical activities, body composition and Glycaemic control among patients with T2D | Randomised controlled trial with three parallel groups (2 interventions + 1 control) | Active control | 8 weeks | To compare the effects of intervention on glycaemic control, blood lipids, body composition, physical activity level, functional capacity, and quality of life with supervised group exercise in patients with type 2 diabetes. | Small sample – underpowered; High attrition rate; Internet-based reporting system and video-watching control system were only used for monitoring the Internet-based exercise training group. There wasn’t a mechanism that would allow the physical therapist to determine whether exercises were completed properly; by adhering to the number of repetitions and periods dietary habits were not monitored, and long-term follow-up for the benefits obtained in the exercise groups was not conducted |
| Diabetes  (n = 10) | [Bendig et al., 2021](https://www.ncbi.nlm.nih.gov/pmc/articles/PMC8273455/pdf/bmjopen-2021-049238.pdf) | An internet-based and mobile-based psychological intervention based on Acceptance and Commitment Therapy -ACTonDiabetes | Reducing diabetes related distress | Randomised controlled trial with two parallel groups | Waitlist/Delayed intervention | 8 weeks | To evaluate the preliminary effect of an intervention on diabetes distress | all data are based on self-report; absence of HbA1c measurement data; effects of waiting for treatment and receiving ACTonDiabetes without guidance (for waistlist control group) could have biased the data; sample was not recruited in a naturalistic setting, thus it may not reflect the real-life uptake of patients in the healthcare system |
|  | [Cohen et al., 2020](https://pubmed.ncbi.nlm.nih.gov/30691328/) | The Health Buddy® electronic device - collects and transmits information about a patient’s chronic condition, including vital signs, symptoms, and behaviours. | To improving diabetes medication adherence, haemoglobin A1C (A1C), and depression | Randomised controlled trial with two parallel groups | Active control | 24 weeks (6 months) | To determine whether a pharmacist-led telehealth disease management program is superior to usual care of nurse-led telehealth in improving diabetes medication adherence, haemoglobin A1C (A1C), and depression scores in patients with concomitant diabetes and depression. | Recruitment bias - must also have a landline phone and a three-prong electrical plug to operate the telehealth equipment; Depression scoring system used was changed midway through the study. |
|  | [Egede et al., 2017](https://pubmed.ncbi.nlm.nih.gov/28581821/) | Technology-assisted case management (TACM) with medication titration by nurses using guideline-based algorithms – included blood glucose monitoring and Individualized care plan | To support diabetes management | Randomised controlled trial with two parallel groups | usual care | 24 weeks (6 months) | To assess the efficacy of an intervention in improving glycemic control in low-income rural adults with poorly controlled type 2 diabetes. | financial constrain caused study to end prematurely; Recruitment bias – within one health system in USA limiting generalizability; Technical glitches of device |
|  | [Franc et al., 2020](https://www.ncbi.nlm.nih.gov/pmc/articles/PMC7757616/) | Telemedicine solution that combines a mobile app for patients with a web portal for health care providers (DIABEO® system) | Improve glycaemic control through Real-time monitoring of basal-bolus insulin therapy as well as therapeutic decision-making by integrating both basal and bolus dose calculation | Randomized controlled, open label multicentre, study with three parallel arms (2 interventions + 1 control) | Usual care | 56 weeks (12 months) | To evaluate the efficacy of the intervention on glycaemic control in conditions close to real life | Low usage rate of DIABEO in pragmatic conditions; assessment of efficacy for glycemic control was performed on a population group basis without considering site specific factors |
|  | [Iversen et al., 2020](https://www.ncbi.nlm.nih.gov/pmc/articles/PMC7580005/) | Follow-up care provided via telemedicine (TM) - interactive web-based ulcer record and a mobile phone enabling counselling and communication between patients and clinician | To support management of diabetes-related foot ulcers (DFU) in outpatient care | Randomized controlled trial with two parallel groups, multicentre | Usual care | 56 weeks (12 months) | To compare changes in self-reported health, well-being and QOL between patients receiving telemedicine follow-up care Vs standard outpatient care | Patients with a diagnosed mental disorder or cognitive impairment, frail elderly unable to consent who might benefit most were excluded – recruitment bias; Inadequate sample size to detect differences on secondary outcome measures; Participants in the study had a relative longstanding diabetes and most likely established self-management routines – difficult for short telemedicine intervention to bring affect |
|  | [Jia et al., 2021](https://www.ncbi.nlm.nih.gov/pmc/articles/PMC8454951/) | Graded ROADMAP app - providing patients with routine contacts, monitoring and evaluation, and lifestyle instructions | To improve glycaemic control in primary care | Randomized controlled trial with two parallel groups – clustered study | Usual care | 56 weeks | To test effectiveness of an intervention on diabetes control | Recruitment was done from registered practice – require caution when extrapolating the findings to a general population with type 2 diabetes; physical and biochemical examinations were conducted using various local resources – possible inherent variations; Less frequency monitoring of blood glucose in control group |
|  | [Mudiyanselage et al., 2018](https://journals.sagepub.com/doi/10.1177/1357633X18775850) | Remote patient monitoring service – a platform TELUS + peripheral equipment | To improve health outcome and quality of life | Randomised controlled trial with two parallel groups | Usual care | 56 weeks (12 months) | To examine the clinical effectiveness and cost-effectiveness of the intervention (pilot) | Small sample not powered to determine a significant difference in the primary outcomes; Data on community-based services such as GP visits and other health service utilisation was not collected – can not tell the confounding effect of these |
|  | [Ramallo-Fariña et al., 2020](https://www.ncbi.nlm.nih.gov/pmc/articles/PMC7669446/) | Web-based complex intervention of knowledge transfer and behaviour modification, informed by conceptual frameworks of behavioural change- with monitoring and personalised feedback massages | To support decision making in management of T2DM | Randomised controlled trial with two parallel groups – multicentre, open label community based | Usual care | 2 years | To assess the effectiveness of internet-based multicomponent interventions to support decision making of all actors involved in the care of patients with T2DM in primary care. | Difficult to obtain a full data set because of the high number of control visits and the duration of follow-up for many patients. – open label study; High turnover among primary care professionals – open label study; Very low baseline HbA1c i.e <7% among 49.4%; Usual care group was not a true control as had care activities going on confounding the study; Not designed to test the efficacy of every component of the complex interventions assessed. |
|  | [Wang et al., 2018](https://pubmed.ncbi.nlm.nih.gov/31368137/) | Continuous diabetes care through mobile app (hand-held clinic, blood glucose monitoring and reminder, dietary recording, exercise guidance, reports etc) | To support continuous care of diabetes out of hospital | Randomised controlled trial with two parallel groups – single blind | Usual care | 24 weeks (6 months) | To explore the clinical effect of continuous care for patients with type 2 diabetes using mobile health application | Selection and recruitment bias – patients residing close to the hospital; Short follow-up duration – long term impact not verified. |
| Cardiovascular (n = 15) | [Ionov et al., 2021](https://www.tandfonline.com/doi/full/10.1080/08037051.2020.1813015) | Blood pressure telemonitoring and remote counselling (BPTM) | To improve blood pressure (BP) control in patients with hypertension (HTN) | Randomised controlled group with two parallel groups (2:1 randomisation) | Usual care | 12 weeks (3 months) | To investigate whether BPTM fits all principles of the value-based approach (clinical and economic effectiveness, improvement in patient-reported outcome/experience measures (PROM/PREM)). | Small sample size; Recruitment/selection bias - digitally literate and technically equipped patients were included; Short duration of the study; Economic analysis based on hypothetical long-term probabilities of an effective but short program |
|  | [Johnston et al., 2016](https://www.sciencedirect.com/science/article/pii/S000287031630062X?via%3Dihub) | Smartphone application ("app") for medication adherence – diary with reminder, interactive patient support portal | To improve treatment adherence and cardiovascular lifestyle in MI patients. | Randomised controlled trial with two parallel groups multicentre | Active control/alternative intervention | 24 weeks | To assess the impact of the app on medication adherence | Because of the nature of the study, it was impossible to blind the intervention to both observer and patient; Objective validation of the self-registered drug use was not possible; Recruitment bias - prerequisite of being smartphone users likely skewed the population to younger MI patients early adopters of tech |
|  | [Peng et al., 2018](https://www.ncbi.nlm.nih.gov/pmc/articles/PMC6392598/pdf/medi-97-e12069.pdf) | Online rehabilitation program - exercise training, follow up an consultation via webcom + WeChat software | To improve quality of life of patients with heart failure | Randomised controlled trial with two parallel groups | Usual care | 24 weeks (6 months) | To examine the effect of intervention on health outcomes in patients with HF | Recruitment bias – sample from a single hospital; Participation required smartphone ownership; Short intervention and follow-up. |
|  | [Kamoen et al., 2020](https://link.springer.com/article/10.1007/s13760-019-01218-z) | Video consultation + educational digital platform | To improve cardiovascular risk factors after ischemic stroke | Prospective Controlled, nonrandomised trial, with a historic control | Historic control | 24 weeks (six months) | To test the efficacy of intervention on cardiovascular risk (pilot) | Recruitment bias - young, motivated patients – naturally on lower risk; Short follow-up period; Limitation of historic control - large amount of missing data in the historical cohort; Data from the historical cohort were collected 6 years prior |
|  | [Koehler et al., 2018](https://www.sciencedirect.com/science/article/abs/pii/S0140673618318804) | Remote patient management intervention | Early detection of signs and symptoms of cardiac decompensation | Randomised controlled trial with two parallel groups | Usual care | 1 year | To investigate the efficacy of intervention on mortality and morbidity in a well-defined heart failure population. | The intervention is made to suit German health-care system with specific emphasis on the interaction between a telemedical centre and local caregivers. Applicability to other care systems will require adaptation |
|  | [Kraai et al., 2016](https://www.sciencedirect.com/science/article/pii/S1386505615300447?via%3Dihub) | Information and Computing Technology-guided-disease-management-system (ICT-guided-DMS) incorporated with Computer Decision Support System (CDSS) | To improve clinical and patient reported outcomes, reduces healthcare costs. | Randomised controlled trial with two parallel groups | active control | 36 weeks (9 months) | To assess the effect of telemonitoring on top of an ICT-guided-DMS with an CDSS in patients with worsening HF on the combined endpoint of death, readmission and HR-QoL, | Under recruited – study underpowered |
|  | [Ling et al., 2021](https://www.ncbi.nlm.nih.gov/pmc/articles/PMC8360129/) | Smartphone devlivered Chronic disease management mode (CDMM) comprising nursing consultations, telephone contact, online WeChat link, health education, and appropriate referrals during hospitalisation and after discharge | To improve patients self-management of hypertension | Controlled, non randomised - Quasi-experimental study | Usual care | 4 weeks | To evaluate the intervention impact on quality of care and clinical outcome | No blinding allocation; Possible bias in interview due to inadequate pyschological knowledge of nurses |
|  | [Lu et al., 2021](https://www.ncbi.nlm.nih.gov/pmc/articles/PMC8477318/) | A computerised clinical decision (impedance cardiography, ICG) support for treatment choices | Improving blood pressure (BP) control in real-world clinical practice. | Randomised control trial with two parallel groups - Pragmatic | Usual care | 12 weeks | To test the effectiveness ofan intervention on improving blood pressure (BP) control in real-world clinical practice. | Inadequate sample size; Relatively short follow-up period; Selection and recruitment bias limiting generalizability of findings - mostly urban, working class Chinese; Did not assess medication compliance among hypertensive patients, which may affect BP values of patients in the two arms |
|  | [McDermott et al., 2018](https://www.ncbi.nlm.nih.gov/pmc/articles/PMC5933394/) | Home-based exercise intervention consisting of a wearable activity monitor and telephone coaching | Improve mobility | Randomised controlled trial with two parallel groups | Usual care | 36 weeks (9 months) | To determine whether a intervention improves walking ability over 9 months in patients with PAD. | Results may not be generalising to all PAD patients; Multiple secondary outcome measures and no adjustment for multiple comparisons; Absence of immediate coach review and feedback of uploaded activity and exercise data may have resulted in an insufficiently potent exercise intervention; 79% adherence to scheduled intervention telephone calls with a coach, maybe insufficient; Insufficient/missing data eg on location of PAD (aorto-iliac vs superficial femoral) |
|  | [Meltzer et al., 2018](https://www.tandfonline.com/doi/abs/10.1080/02687038.2017.1355440?journalCode=paph20) | Post stroke speech telerehabilitation (clinician guided computer-based therapy, using WebEx videoconference) | Improved post-stroke language and communication | Randomised controlled trial with two parallel groups – non inferiority | Usual care | 12 weeks | To evaluate the effectiveness of telerehabilitation | Small sample – underpowered |
|  | [Sarfo et al., 2019](https://pubmed.ncbi.nlm.nih.gov/30465630/) | Blue-toothed BP device and smartphone with an App for monitoring BP measurements and medication intake | Improving blood pressure (BP) control | Randomized controlled trial with two parallel groups - clustered study | Usual care | 36 weeks (9 months) | To assess the effect if the intervention on PB control (pilot) | All participants at screening visit met systolic BP entry criterion (BP ≥140 mmHg), but half in each arm had systolic BP < 140 mmHg at the enrolment visit - this might have diluted the effect of the intervention |
|  | [Thielbar et al., 2017](https://ieeexplore.ieee.org/document/7470432) | An innovative voice and electromyography-driven actuated (VAEDA) glove for post stroke hand rehabilitation | Hand-focused occupational therapy to Improve motor control | Randomised controlled trial with two parallel groups | Usual care | 6 weeks | To examine the therapeutic benefits of the device | Inherent differences on motor strengths between comparison groups; Gloves weight increase resistance to exercise might compromise true results |
|  | [Thompson et al., 2021](https://www.ncbi.nlm.nih.gov/pmc/articles/PMC7876856/pdf/PRP2-9-e00710.pdf) | Telehealth for optimisation of medical therapy | To increase adherence to medication | Randomised controlled trial with two parallel groups | Usual care | 12 weeks | To evaluate efficacy of the intervention in self reported medication adherence | Technical limitations – measurement of drug at time of testing doesn’t guarantee the adherence, could aswell be the reflection of single dose taken immediately before test. |
|  | [Wentink et al., 2016](https://pubmed.ncbi.nlm.nih.gov/27184585/) | Computer-based brain training programme on cognitive functioning, QoL and self-efficacy, (Lumosity Inc.®) | Improve quality of life, cognitive and functionality of stroke patients | Randomised controlled trial with two parallel groups | Usual care | 8 weeks | To determine the effect of an intervention on cognitive functioning, QoL and self-efficacy | Recruitment bias – based on subjective cognitive impairment criteria, computer literate only; Small sample - underpowered |
|  | [Paruchuri et al., 2021](https://pubmed.ncbi.nlm.nih.gov/34657825/) | a smartphone app-based platform for hospitalized patients receiving percutaneous coronary intervention (PCI) which included education, tracking, reminders and live health coaches | To bridge high risk care gaps for post-percutaneous intervention and improve adoption of guideline-supported therapies | single-arm open-label with 1:3 propensity-matched historical controls (Pilot) | Historic control | 12 weeks | To evaluate feasibility and efficacy | Recruitment bias – ownership of smartphone and ability to use app; Use of historical controls may lead to imbalance of unavailable phenotypes or systematic healthcare changes; short duration and small sample size |
| **DH aiming at aiding disease self-management (n = 45)** | | | | | | | | |
| Cancer  (n = 10) | [Absolom et al., 2021](https://pubmed.ncbi.nlm.nih.gov/33417506/#:~:text=Phase%20III%20Randomized%20Controlled%20Trial%20of%20eRAPID%3A%20eHealth,treated%20with%20curative%20intent%2C%20without%20increasing%20hospital%20workload.) | Electronic patient self-Reporting of Adverse-events; Patient Information and aDvice (eRAPID) system combine secure online symptom self-reporting with an innovative bespoke clinical algorithm, generating automated severity-based advice to patients | To support self-management or prompt hospital contact for chemotherapy patients | Randomised controlled trial with two parallel groups | Usual care | 18 weeks | To evaluate the impact of eRAPID on symptom control, healthcare use, patient self-efficacy, and quality of life (QOL) in a patient population treated predominantly with curative intent. | The chosen primary outcome FACT-PWB scale included a combination of key treatment-related symptoms and functional items, and therefore, there was a possibility that improvements in symptoms might be diluted by limited changes in functions; Single center trial pragmatic nature; As clinicians saw patients; In both arms, the symptom reports may have sensitized providers to interact differently and conduct in-depth symptom assessments with UC patients |
|  | [Børøsund et al., 2020](https://pubmed.ncbi.nlm.nih.gov/32243717/) | An app‐based cognitive‐behavioral stress‐management intervention program for cancer survivors - StressProffen TM© | Record and report daily functional activity or symptoms with indication of severity | Randomised controlled trial with two parallel groups | Usual care | 12 weeks (3 months) | To evaluate the preliminary efficacy of an application (app)-based cancer stress-management intervention on cancer patients | Recruitment bias – motivated cancer survivor , predominantly breast cancer; Unreliability of self-reported baseline data; Study likely to be underpowered; Low response; Assessment of mood was done by questionnaire only – interview could have given different outcome |
|  | [Egbring et al., 2016](https://www.ncbi.nlm.nih.gov/pmc/articles/PMC5030453/) | Novel open-source mobile and Web app to record daily functional activity and adverse events. | To Improve symptoms, physical strength and quality of life | Randomized controlled trial with three parallel groups. | Usual care | 6 weeks | To evaluate the intervention on patient functional activity | Inadequate sample size; Intervention was unblind to participants and physician, might have impacted responses to questionnaire; Different chemotherapeutic regimens may cause different adverse events and functional activities |
|  | [Galiano-Castillo et al., 2016](https://pubmed.ncbi.nlm.nih.gov/27332968/) | Information on breast cancer and tailored exercise program telerehabilitation program was implemented using the e-CUIDATE system consists of a public interface and a separate private interface | To support self-management for adverse drug reactions eg anxiety and depression | Randomised controlled trial with two parallel groups | usual care | 24 months (6 months) | To investigate the effectiveness of an intervention after 8-week and 6 months of follow-up in BC survivors. | Recruitment bias - eligibility criteria as well as the mean age of our sample may limit the generalization of results; Short duration intervention |
|  | [Handa et al., 2020](https://pubmed.ncbi.nlm.nih.gov/32201165/) | Breast cancer patient support system (BPSS) application (app) – provides self-care information and chronologically and quantitatively records patients' subjective and objective symptoms during breast cancer chemotherapy | To support symptoms self-management of breast cancer patients receiving systemic therapy | Randomised controlled trial with two parallel groups | Active control | 12 weeks | To examined effectiveness of the BPSS app in supporting patients undergoing chemotherapy. | Small sample size; Conducted at a single centre; Short follow-up duration |
|  | [Kuhar et al., 2020](https://pubmed.ncbi.nlm.nih.gov/32427567/) | mPRO Mamma mobile app - quick daily recording of symptoms and symptom severity | Real-time symptom assessment and management during radiotherapy for localized prostate cancer | Controlled, non-randomized study - two groups | Usual care | No data | To evaluate whether use of an app for symptom management was associated with any change in patient quality of life or use of health resources | Not randomised; groups in the study differed with respect to type of breast surgery, quality of life score, and summary score at baseline ( the results were adjusted for these differences using appropriate statistical methods); seasonal differences affecting patient well-being since patients were enrolled at different times of the year; not all symptoms that can arise during the systemic therapy can be assessed with the QLQ C-30 and BR-23 questionnaires |
|  | [Sundberg et al., 2017](https://www.ncbi.nlm.nih.gov/pmc/articles/PMC5445148/) | Smartphones and tablets application (Interaktor) for early detection, reporting and management of symptoms | To identify symptoms early and in real time, and to support symptom management. | Controlled, non randomised – two groups | Historic control | 12 weeks (3 months) | To evaluates the effect an intervention on symptom burden and quality of life | Potential confounders not controlled due to the nature of the design - historical control; Comparable groups differ educational level confounding self-reported QoL |
|  | [Sundberg et al., 2021](https://pubmed.ncbi.nlm.nih.gov/32811748/) | The interactive app (Interaktor) for self- reporting and management of symptoms – connect symptoms self-report with web-based clinic monitoring interface, tailored advice | Improve cancer and Treatment Distress (CTXD), depression, physical functioning and quality of life | Controlled, non randomised - Quasi-experimental | Historic control group | 12 weeks (3 montsh) | To determine the effect of Interaktor app for symptom management on health literacy levels and self-care ability in men with prostate cancer during radiotherapy | None reported |
|  | [Syrjala et al., 2018](https://pubmed.ncbi.nlm.nih.gov/29730827/) | Individualized online information resources and telehealth call for post operative symptoms management program (INSPIRE) | To support self-management and health quality of life by monitoring health-related quality of life (HRQOL) and cancer-generic and tumour-specific symptom | Randomised controlled trial with three parallel groups (2 interventions + 1 control) | Waitlist control/Delayed intervention | 24 weeks (6 months) | To examine the efficacy on intervention | Recruitment bias – not diverse sample hence not representative (single centre, internet access) |
|  | [Van der Hout et al., 2020](https://pubmed.ncbi.nlm.nih.gov/31838009/) | A web-based eHealth application (Oncokompas) for self-management supports for cancer survivors through symptom monitoring and personalised care support | To support self-management or prompt hospital contact | Randomised controlled trial with two parallel groups | wait list/delayed intervention | 24 weeks (6 months) | To assess the efficacy, reach, and usage of an intervention | Recruitment bias – access to internet; Complex intervention but the study was not powered to account for the variation in outcome measures – subject to random error; High attrition on control group |
| Diabetes mellitus  (n = 22) | [Baldwin et al., 2020](https://www.ncbi.nlm.nih.gov/pmc/articles/PMC7738252/) | Automated and self-guided interactive web-based mental health program (myCompass) | Self-monitoring and reporting cognitive behaviour, plus educational support | Randomized controlled trial with two parallel groups | Active control (a healthy lifestyle module without CBT content) | 56 weeks (12 months) | To establish the impact of intervention on daily functioning of adults with T2D and depressive symptoms | High attrition of participants with severe distress and anxiety symptoms [Retention strategies for future studies may need to vary across study phases, and the impact of this apparently biphasic pattern of attrition could be taken into account when analyzing results] |
|  | [Anzaldo-Campos et al., 2016](https://pubmed.ncbi.nlm.nih.gov/26914371/) | Diabetes self-management educational program with MyGlucoHealth glucose meter (Entra Health Systems, San Diego) connected to a 3G-enabled cell phone | Improving clinical and other self-report outcome in patients with type 2 diabetes in Mexico. | Randomized controlled trial, open-label with three parallel groups (2 interventions + 1 control) | Usual care | 40 weeks (10 months) | To evaluated if the adapted intervention for the Mexican population, with and without mobile technology, was effective in a “real-world” environment (i.e., under routine practice conditions with evolving management approaches), compared with usual clinical care, at improving clinical and self-report outcomes in patients with type 2 diabetes in Mexico. | Limited lab tests – supplies shortage; Recruitment bias – sample unrepresentative of general population; mobile technology tools were actively only offered to the participants for the first 2 months only – might have cause low compliance due to tech unavailability; Incomplete dataset due to participants entering and exiting the system |
|  | [Baron et al. 2017](https://pubmed.ncbi.nlm.nih.gov/26880694/) | Diabetes self-management program with measuring devices connect with mobile phone software for recording and sharing data | Glycaemic control | Randomised controlled trial with two parallel groups | Usual care | 36 weeks (9 months) | To determine the effects of intervention on HbA1c and other clinical and patient-reported outcomes | Inadequate sample size - under recruiting; Low response rate limiting generalisability of findings; Usage data Vs treatment effectiveness was not examined |
|  | [Baron et al., 2017](https://journals.sagepub.com/doi/10.1177/1357633X16655911) | Mobile-phone based home telehealth (MTH)Disease management education and recording of clinical data | To improve self-efficacy, illness beliefs, and diabetes self-care | Randomized controlled trial with two parallel groups | Usual care | 36 weeks (9 months) | To investigate the effects of an intervention in self-care behaviour of people with diabetes. | Small sample size; Short follow-up period; Important confounding factors in Social Cognitive theory not accounted for |
|  | [Boels et al., 2019](https://www.ncbi.nlm.nih.gov/pmc/articles/PMC6196442/) | Smartphone app, designed to trigger diabetes self-management (TRIGGER app). - messages regarding dietary habits, physical activity, prevention of hypoglycaemia, and glucose variability | To promote self-management of diabetes | Randomised controlled trial with two parallel-groups, multicentre | Usual care | 24 weeks (6 months) | To evaluate the effectiveness of diabetes self-management education via a smartphone app in T2DM patients on insulin therapy. | Patients in the control group might be using other diabetes apps during the study; Since it’s a real-life pragmatic trial; in real-life low compliance is expected - non-use or infrequent use of a smartphone app |
|  | [Chiu et al., 2020](https://www.ncbi.nlm.nih.gov/pmc/articles/PMC7298636/) | Social and communication app (LINE) for disease self-management | To improve depressive symptoms and glycemic control. | Controlled, non-randomised 3-arms (quasi experimental?) – 2 interv + 1 control | Usual care | 12 weeks | To test the employment of a free and widely used to help older adults with diabetes manage their distress and glycaemic control. | Inadequate sample size; No randomisation; No evaluation of changes in behaviour-related variables |
|  | [Deshpande et al., 2020](https://pubmed.ncbi.nlm.nih.gov/32319791/) | Smartphone-based AID application platform, the interoperable artificial pancreas system (iAPS) - r uns on an unlocked smartphone and interfaces wirelessly with leading CGMs, insulin pump devices, and decision-making algorithms. | Improving glycaemic control for people with T1D | Randomised controlled with two cross-over groups | Usual care | 4 weeks | To compare postprandial, as well as overall, glucose control related to AID use versus conventional therapy | The study was conducted on a small number of subjects with tight baseline glycemic control, this limits scope of improvement with AID-based control and overall generalizability; As the meal challenges were conducted at home in free living conditions, we cannot completely isolate the effect of study meal from other causes of glycemic disturbances (meals or otherwise). |
|  | [Garnweidner-Holme et al., 2020](https://www.ncbi.nlm.nih.gov/pmc/articles/PMC7673980/) | Smartphone app (Pregnant+) with targeted dietary information and blood glucose monitoring for management of Gestational diabetes mellitus | To support self-management of GDM through providing tailored information on diet, physical activity, breastfeeding, and GDM, and automatically transfer or manually record blood glucose levels from a glucometer to the smartphone. | Randomised controlled trial with two parallel groups – multicentre | Usual care | 36 weeks | To determine the effect of intervention on dietary behavior and blood glucose on women with Gestational diabetes mellitus | Did not have access to usage logs because of technical problems – hence usage not accessed; Patients participating in a study will often experience an effect even when not receiving the intervention—the Hawthorne effect; The data for this study were derived from self-completed questionnaires, which include the possibility for recall bias; Unreliability of FFQ - covered only selected aspects of the overall diet. Thus results should not be interpreted as an absolute measure of dietary change |
|  | [Gimbel et al., 2020](https://pubmed.ncbi.nlm.nih.gov/32329438/) | A mobile health care accessed mobile phone/tablets with Bluetooth-enabled medical devices and portal for care information/messages | To enhance patient activation and self-management of T2D | Randomised controlled trial with two parallel groups, multicentre | Active control/alternative intervention | 56 weeks (12 months) | To evaluate the feasibility of an intervention to enhance Patient Activation and Self-Management Activities | Control group might have accessed intervention; Uncontrolled food intake and daily activities outside the research sites |
|  | [Huang et al., 2021](https://pubmed.ncbi.nlm.nih.gov/33771645/) | Gestational diabetes management program via WeChat (involve patients sharing photos of their meals, exercise and records of blood glucose control and weight gain and receive real-time guidance) | Supporting self-management of gestational diabetes | Randomised controlled trial with two parallel groups – multicentre | Usual care | 28 weeks | To investigate the effects of mobile health based peripartum management of gestational diabetes mellitus (GDM) on postpartum diabetes and factors associated with postpartum diabetes. | The sample size calculation was based on the glycemic control rate rather than the incidence of postpartum T2DM -might be an underestimated sample size for postpartum outcomes; Use of WeChat groups instead of an APP designed to support 1-to-1 management of GDM; 8.92% of women were enrolled at the 29 to 30+6 gestational week - the intervention period may be too short to see the significance of effectiveness. |
|  | [Kerfoot et al., 2017](https://pubmed.ncbi.nlm.nih.gov/28790131/) | Mindfulness gaming for diabetes management delivered via email or mobile aoo | Glycaemic control | Randomised controlled trial with two parallel groups | Active control (game delivered via booklet) | 56 weeks (12 months) | To investigate the long term impact of intervention on glycaemic control | Recruitment bias - Enrolled patients with access to internet and email; Short duration for long term effect evaluation; Participants’ engagement with paper-based material and influence of outcome were not evaluated |
|  | [Lee et al., 2020](https://www.ncbi.nlm.nih.gov/pmc/articles/PMC7532462/) | mHealth-based diabetes self-management education, consisting of the mobile app Healthynote (for Android; CVnet Co) and regular individualized feedback messages from health care professionals regarding their diabetes management | BP and Glycaemic control; Increase physical functioning; Improve health behaviour and quality of life | Randomized controlled trial with two parallel groups, open label | Usual care | 24 weeks (6 months) | To evaluate whether implementing mHealth-based diabetes self-management education could improve diabetes self-management and glycemic control and enhance patient quality of life. | Small sample size; Recruitment bias – limited to android users; Open label – study environment not controlled subject to confounders; Technical faulty limited assessment of data usage |
|  | [Lemelin et al., 2020](https://www.liebertpub.com/doi/10.1089/dia.2019.0324) | Automated personalised guidance on diet, physical activities, and medication. Monitoring of health outcomes | Self-management of Diabetes | Controlled trial, non-randomised, noninferiority study | usual care | 24 weeks (6 months) | To evaluate the impact of telehomecare (THC) use on clinical efficacy, nursing interventions, and medical visits | Lack of formal randomisation; No baseline glycaemic data; No evaluation of engagement with intervention |
|  | [Mayberry et al., 2021](https://pubmed.ncbi.nlm.nih.gov/32706852/) | Mobile phone-delivered tailored messages for diabetes self-management (FAMS) | To improve Medication adherence and Glycaemic control | Randomised controlled trial with three parallel groups (2 interv + 1 control) | Active control | 24 weeks (6 months) | To evaluate the acceptability and impact of an intervention in self-efficacy and self-care of adults with T2D | Measure of adherence (text message) had inherent limitation as also acted as reminder; Study was not able to assess the individual effect of multicomponent of the intervention |
|  | [Montero et al., 2021](https://www.ncbi.nlm.nih.gov/pmc/articles/PMC8582211/) | A novel BGM system which auto-transfers near real-time FSBG data to a cloud-based dashboard using cellular networks | To support Self-monitoring of blood glucose | Controlled non-randomized trial with propensity-matched controls. | Historic control | 12 weeks | To evaluate the impact of SMBG utility and frequency on glycemic outcomes using a novel BGM system | Selection bias – predominantly black aged 40 – 60, with uncontrolled T2DM; Did not attempt to identify the impact of individual components of this multifaceted intervention on outcomes |
|  | [Nelson et al., 2017](https://www.ncbi.nlm.nih.gov/pmc/articles/PMC5176095/) | MEssaging for Diabetes (MED) SMS and interactive voice response (IVR) | To improve medication adherence and glycaemic control | Controlled, non-randomised quasi-experimental | matched, archival control group | 12 wees | To test the impact of intervention on glycaemic control | Recruitment bias – from single centre limiting generalizability; High attrition/loss to follow up; No true control; No randomisation |
|  | [Sun et al., 2019](https://www.ncbi.nlm.nih.gov/pmc/articles/PMC6682265/) | Mobile phone–based telemedicine apps for Diabetes management consisting of medication, diet, and exercise guide | To support diabetes self-management | Randomised controlled trial with two parallel groups | usual care | 24 weeks (6 months) | To investigate the efficacy (glycaemic control, meds adherence) and safety (adverse events occurrence) of an intervention for management of older Chinese patients with T2DM | Confounding effect of baseline characteristics not considered – medical history, smoking, living environment etc |
|  | [Sunil Kumar et al., 2020](https://www.sciencedirect.com/science/article/pii/S1871402120302733?via%3Dihub) | Mobile application for medication management and lifestyle modification for patients with type 2 diabetes | To improve the quality of life of patients | Randomized controlled trial of two parallel groups | Usual care | 24 weeks (6 months) | To assess the effect of smartphone-based lifestyle modification intervention in the quality of life of patients with type 2 Diabetes | No power calculation for sample size. The number was conveniently decided on. |
|  | [Yaron et al., 2019](https://pubmed.ncbi.nlm.nih.gov/30783823/) | Blood glucose self-monitoring and remote consultation, glucometer and insulin pump linked with clinic system | Glycaemic control | Randomised controlled trial of two parallel groups | usual care | 56 weeks (12 months) | To examine the effectiveness and safety of intervention | Inadequate sample size due to drop out; Lack of continuous glucose monitoring (CGM) data |
|  | [Yasmin et al., 2020](https://www.ncbi.nlm.nih.gov/pmc/articles/PMC7282058/) | Mobile phone-based health intervention - a patient reminder system through interactive voice calls to support self-care | To increase patient adherence to treatment and improve the disease outcomes | Randomised controlled trial with two parallel groups – with mixed-method approach | Usual care | 56 weeks (1 year) | To investigated how mobile phone-based health intervention could increase patient adherence and thereby improve the disease outcomes for diabetes type 2 in Bangladesh. | Recruitment bias: patients from a single tertiary hospital ; Predominantly female; Unreliability of self-reporting data including adherence |
|  | [Zhou et al., 2016](https://www.sciencedirect.com/science/article/abs/pii/S0168822716300523) | Smart phone-based diabetes management application, Welltang – education, guidance + glucose monitoring functions | To improve glycaemic control | Randomised controlled trial with two parallel groups | Usual care | 12 weeks | To evaluate the impact of the intervention on glycated hemoglobin (HbA1c) | Small sample size; Short follow up duration; multi-component intervention – could not quantify contribution of individual elements |
|  | [Batch et al., 2021](https://pubmed.ncbi.nlm.nih.gov/33470947/) | A self-guided mobile app for diabetes education (Time2Focus app; MicroMass Communications Inc, Cary, NC), which utilizes evidence-based content and gamification to deliver an interactive learning experience. | To improve HbA1c, and patient confidence and skills in effectively managing diabetes | Single arm followed up with non-responders used as comparator (Pilot study) | Non responders | 12 weeks | To test a real-world deployment and efficacy of the app on HbA1c and change in self-reported health behaviours | a small cohort, the use of naturally occurring HbA1c as the primary outcome, the short duration of the study, and lack of randomization. High allow completion rate (23.8)H |
| Cardiovascular  (n = 9) | [Engelen et al., 2020](https://www.ncbi.nlm.nih.gov/pmc/articles/PMC7414414/) | web-based self-management program contained 6 modules (coping with CVD, setting boundaries, lifestyle, healthy nutrition, being physically active, interaction with health professionals) | To increase self-management behaviour by tailoring to the perceived problems and (support) needs of patients after CVD | Randomised controlled trial with two parallel groups -explorative | Usual care | 56 weeks (56 months) | To evaluate the potential effectiveness and effect sizes of the Vascular View program and identifying outcome measures most likely to capture the potential benefits. | More patients in the intervention compared with the control group were lost to follow-up; Research was conducted in the outpatient clinic of a university hospital in which high quality of CVD care is already delivered, and therefore it may be harder to achieve improvement; Possible recruitment bias – those already interested in self-management |
|  | [Jung et al., 2017](https://journals.sagepub.com/doi/10.1177/1357633X15621467?url_ver=Z39.88-2003&rfr_id=ori:rid:crossref.org&rfr_dat=cr_pub%20%200pubmed) | In-class educational phase, community-based eHealth monitoring, and monthly telephone counselling | To support elderly living alone in the community, manage hypertension | Controlled non randomised quasi-experimental ? | Active control/alternative intervention | 24 weeks | To examine the effect of the intervention on BP control (pilot) | No randomisation; Small sample size |
|  | [Pekmezaris et al. 2019](https://pubmed.ncbi.nlm.nih.gov/30418101/) | Telehealth self-monitoring of vital function and remote consultation – monitoring devices connected wirelessly with phone and care provider system | Reducing admission to emergency department; Improved quality of life | Randomised controlled trial with two parallel groups | Active control/Alternative intervention | 90 days | To assess of intervention on health care utilization and QoL | Single centre study, results may not be generalizable to other underserved populations living in different settings; Advances in technology outpaced the evaluation and reporting of efficacy. The tech evaluated was already outdated when publishing this study; Many statistical tests performed, increasing the probability of finding significance |
|  | [Still et al., 2021](https://www.ncbi.nlm.nih.gov/pmc/articles/PMC8646778/) | Analytic and emotional technology-based support (web-based education + BP monitoring device + medication management app) | Self-managing hypertension and psychological well-being | Randomized controlled trial of two parallel groups (pilot) | Active control (with all elements of intervention except emotional support) | 12 weeks | To evaluate the effects of intervention on BP, QOL and psychological health | Inadequate sample size(pilot); Convenient sampling procedure; Short duration of study, hence long term effects of intervention unknown |
|  | [Tiede et at., 2017](https://journals.sagepub.com/doi/10.1177/1357633X16668436?url_ver=Z39.88-2003&rfr_id=ori:rid:crossref.org&rfr_dat=cr_pub%20%200pubmed) | Telephone-based health coaching (TBHC) – tailored to individuals need | To support disease self-management through telephone health coaching | Randomised controlled trial with two parallel groups | Usual care | 3 years | To examine the long-term effectiveness of a TBHC programme especially tailored for HF | Unreliability of self-reported data; Recruitment bias – volunteers could be different from no participants; Missing measurements of baseline data hence could not compare baseline status between groups; Many potentially confounding variables were not taken into consideration, e.g. medication and comorbidity. |
|  | [Wolf et al., 2016](https://www.ncbi.nlm.nih.gov/pmc/articles/PMC4783584/) | eHealth Diary and Symptom-Tracking Tool Combined With Person-Centered Care for patients diagnosed with Acute Coronary Syndrome | To improve Self-Efficacy After a Diagnosis of Acute Coronary Syndrome | Randomised controlled trial with two parallel groups | Usual care | 24 weeks (6 months) | To investigate the effect of an eHealth diary and symptom-tracking tool in combination with PCC for patients with acute coronary syndrome (ACS). | Possible recruitment bias – highly motivated individuals; No data was collected on the engagement with the eHealth tool as part of the follow-up visits at the outpatient clinic or in primary care |
|  | [Yu et al., 2020](https://www.sciencedirect.com/science/article/abs/pii/S000287032030199X?via%3Dihub) | Smartphone application (Heart Health Application) - medication reminders, cardiac health education, health questionnaire and feedback | To improve medication adherence | Randomized controlled trial with two parallel groups - clustered | Usual care | 24 weeks (6 months) | To evaluate the effectiveness and feasibility of using an intervention to improve medication adherence in patients after CABG. | Participants' baseline measures of adherence not measured; self-reported questionnaires are subject to social desirability bias and may overestimate true adherence; short-term follow-up |
|  | [Zha et al., 2020](https://journals.sagepub.com/doi/10.1177/0193945919847937?url_ver=Z39.88-2003&rfr_id=ori:rid:crossref.org&rfr_dat=cr_pub%20%200pubmed) | MHealth for self-monitoring and self-management of BP – BP monitor synchronised via Bluetooth with mobile app | To support self-management of hypertension | Randomised controlled trial with two parallel groups | Usual care | 24 weeks (6 months) | To examine the impact on intervention on BP, self-efficacy, and quality of life | Study did not consider pharmacological treatment or other clinical interventions received; Unreliability of self-measuring and reporting of BP |
| Multiconditions  (n = 4) | [Araya et al., 2021](https://www.ncbi.nlm.nih.gov/pmc/articles/PMC8114139/) | Low-intensity, behavioural activation digital intervention delivered via smartphone (CONEMO) | To reduce depressive symptoms | Randomised controlled trial with two parallel groups - Clustered | Enhanced Usual care | 6 weeks | To investigate the effectiveness of a digital intervention in reducing depressive symptoms among people with diabetes and/or hypertension. | Enhanced usual care included a safety net for high-risk participants for ethical reasons, which likely improved outcomes in the control group; Digital intervention used nurses to support patient app use - findings might not be generalized to fully automated deployment; Study cannot distinguish the relative contribution of the main components (ie, the app and nurses) to improve adherence or outcomes |
|  | [Li et al., 2021](https://www.ncbi.nlm.nih.gov/pmc/articles/PMC8354275/#:~:text=The%20Perx%20trial%20was%20a%20randomised%20controlled%20trial,February%202018%20and%20ended%20on%207%20January%202019.) | Medication adherence app based on behavioural science, gamification with customisable medication regimen details and reminders, educational messages about disease management and healthcare visit prompts | To improve medication adherence | Randomised controlled trial with two parallel groups | Usual care | 56 weeks (12 months) | To determine the impact of the app on medication adherence | Baseline measures of adherence were not assessed; Returning medication bags to the research nurse at study visits may have promoted self-monitoring in both groups confounding the impact of intervention; Participants had good baseline glycaemic control, potentially reducing the generalisability of findings; The app was exclusively online, limiting accessibility when participants were not connected to the internet; People who stayed in the Perx arm or left the control arm after the initial visit potentially had predisposition towards using phones or apps; it was possible for some participants to reveal their group allocation to their healthcare providers |
|  | [Or et al., 2020](https://www.ncbi.nlm.nih.gov/pmc/articles/PMC7148548/) | Technological surrogate nursing (TSN) with interactive access to educational resources for management of T2DM and hypertension and health monitoring. | Supporting self-management of diabetes and Hypertension control | Randomized controlled trial, with two parallel groups | Usual care | 24 weeks | To test and demonstrate the effectiveness and safety of prototype TSN supplied to patients with the typical complex chronic disease of coexisting type 2 diabetes and hypertension | Recruitment bias – long-term patients with stable conditions; Short duration of the study; Confounding effect of medication used not assessed |
|  | [Wu et al., 2017](https://pubmed.ncbi.nlm.nih.gov/28544230/) | Integrated cardiac‐diabetes self‐management program incorporating telephone contact and text message follow-up (T-CDSMP) | Improve Self‐management practice and self‐efficacy | Randomised Control Trial with two parallel groups – blocked by country | Usual care | 4 weeks | To evaluate the short-term effectiveness of the intervention and investigate the interaction effect between intervention, setting and timing of the assessment. | No evaluation/account of influence of ethnicity, country, time on outcome measures; Unreliability of patient-reported outcome measures  - robust objective physiological measures or clinical outcome indicators could be more helpful; Only four weeks of follow-up and one hospital in each country |
| **DH for Preventive, behavioural change (n = 28)** | | | | | | | | |
| Cancer  (n = 8) | [Chung et al., 2020](https://www.ncbi.nlm.nih.gov/pmc/articles/PMC6966488/pdf/fonc-09-01505.pdf) | Online physical activity program - mobile app-based community with health information on diet and physical activities | To promote behaviour, change by Increasing physical activities; Reduce mental distress | Controlled trial, non- randomised + a separate historic data for further comparison | Wait list/delayed intervention | 12weeks | To investigate the effect of a mobile app-based community on enhancing PA and decreasing distress in breast cancer survivors | Lack of randomisation; short term effects measured – long effect unknown; No validation status of distress scoring system |
|  | [Buscemi et al., 2020](https://www.ncbi.nlm.nih.gov/pmc/articles/PMC7374026/) | Two behavioural lifestyle change interventions delivered by smartphone: My Health, (focused on diet and physical activity), and My Guide, (focused on psychosocial functioning) | To improve dietary intake and physical activities | Randomised controlled trial with two parallel groups | Active control/Alternative intervention | 8 weeks | To compare the effectiveness of two intervention on dietary and physical activity of cancer survivors | Did not utilize gold standard measures for diet and physical activity due to participant burden, budgetary restrictions, and risk for drop out; Both interventions included content on diet and physical activity. limiting ability to detect differences between groups |
|  | [Golsteijn et al., 2018](https://ijbnpa.biomedcentral.com/articles/10.1186/s12966-018-0734-9#auth-Rianne_Henrica_Johanna-Golsteijn) | Tailored physical activity advice, via an interactive website and with printed materials OncoActive | Improve physical functioning, distress, fatigue and quality of life | Randomised control trial with waitlist control | wait list/delayed intervention | 24 weeks (6 months) | To assess the efficacy of an intervention in (four subgroups of) prostate and colorectal cancer survivors. | Small sample – low proportion on eligible patients available i.e adjuvant treatment |
|  | [Kanera et al., 2017](https://www.ncbi.nlm.nih.gov/pmc/articles/PMC5303303/) | A fully automated web-based cancer aftercare intervention consisting of a lifestyle changes modules (PA, diet, smoke sensation, anxiety and depression control) | To behaviour and lifestyle - physical activity and Vegetable consumption | Randomised controlled trial with two parallel groups | usual care | 56 weeks (12 months) | To evaluates the 12-month effects of an intervention on moderate physical activity and vegetable | Unreliability of Self-reported outcomes; Recruitment bias - Sample unrepresentative of general cancer population |
|  | [Lozano-lozano et al., 2020](https://pubmed.ncbi.nlm.nih.gov/31454561/) | BENECA mHealth lifestyle application - Behaviour and lifestyle change program | To improve physical activities and Quality of life | Randomized controlled trial with two parallel groups | Active control | 24 weeks (6 months) | To examine the clinical efficacy of the mHealth intervention combined with a supervised rehabilitation program on quality of life (QoL) and functional outcomes of breast cancer survivors. | One group did not have the active presence of a therapist, the impact of the therapist's attention cannot rule out; Inadequate sample sizes to allow comparisons between patients and groups to identify moderators of the treatment effect; No true control |
|  | [Uhm et al., 2016](https://link.springer.com/article/10.1007/s10549-016-4065-8) | Smartphone exercise application called Smart After Care (BIT Computer Co., Ltd., Seoul, Korea) and an InBodyBand pedometer (InBody Co., Ltd., Seoul, Korea) | To improve physical functioning and quality of life of breast cancer survivor | Quasi-randomized multicentre trial | Active control/alternative intervention | 12 weeks | To investigate and compare the effects of intervention on physical function and quality of life | Short duration – so long-term impact unkown; The outcomes of mHealth may be different for the patients who utilize the application during treatment or with other types of cancer |
|  | [Willems et al., 2016](https://onlinelibrary.wiley.com/doi/10.1002/pon.4113) | Web-based computer-tailored Psychosocial therapy and lifestyle training modules - Kanker Nazorg Wijzer | To support cancer survivors with managing psychosocial and lifestyle-related issues | Randomised controlled trial with waitlist control | wait list/delayed intervention | 56 weeks (12 months) | To evaluate the short-term effectiveness on quality of life, anxiety, depression, and fatigue | Group contamination risk due to wait list/delayed intervention control; Recruitment bias – predominantly breast cancer, high scores of baselines psychosocial wellbeing, young age; Unreliability self-administered questionnaire |
| Diabetes mellitus  (n = 6) | [Coombes et al., 2022](https://journals.lww.com/acsm-msse/Fulltext/2022/01000/Personal_Activity_Intelligence_e_Health_Program_in.5.aspx) | Personal Activity Intelligence (PAI) is a new metric that uses the heart rate response to PA to inform the user as to whether they are doing enough PA | To reduce the risk of premature mortality through promoting physical activities | Randomised controlled trial with two parallel groups (pilot) | Active control | 12 weeks | To reduce the risk of premature mortality through promoting physical activities | small sample size (pilot) |
|  | [Hilmarsdóttir et al., 2021](https://www.ncbi.nlm.nih.gov/pmc/articles/PMC8442170/) | A healthy-lifestyle-supporting smartphone application (SidekickHealth) - personalized recommendations and education about healthy lifestyles | To the impact of complementing standard care with a general lifestyle program (SidekickHealth) through a smartphone app on health outcome of people with T2DM, at a hospital-based endocrinology outpatient clinic. | Randomised controlled trial with two parallel groups | Usual care | 24 weeks | To the impact of complementing standard care with a general lifestyle program (SidekickHealth) through a smartphone app on health outcome of people with T2DM, at a hospital-based endocrinology outpatient clinic. | Small sample size due to under-recruitment (resource constrains); No follow-up after the intervention, any long-term effects are unknown. |
|  | [Höchsmann et al., 2019](https://pubmed.ncbi.nlm.nih.gov/30758293/) | Behaviour change technique-based smartphone game | To motivate increase in physical activities | Randomised controlled trial with two parallel groups | Active control/Alternative intervention | 24 weeks | To motivate increase in physical activities | no objectively measured record of any additional PA beyond the phone-recorded PA and outside the structured intervention |
|  | [Lim et al., 2021](https://pubmed.ncbi.nlm.nih.gov/34081137/) | Diabetes Lifestyle Intervention (diet and physical activities) delivered using mobile app (D’LITE) | To achieve individualised goal on caloric intake and physical activity through coaching and monitor/tracking | Randomized controlled trial with two parallel groups - Multiple centres | Active control | 12 weeks (3 months) | To achieve individualised goal on caloric intake and physical activity through coaching and monitor/tracking | Recruitment bias sample might not be fully representative of the target population; Participants with depression were excluded due to the confounding effect of antidepressants; Recruited smartphone users who were literate in English; Unreliability of self-reporting and lacked a validated measure; Contributions of different app components on outcome measures not evaluated |
|  | [Plotnikoff et al., 2017](https://pubmed.ncbi.nlm.nih.gov/28887192/) | Innovative, lifestyle program known as ‘eCoFit’, which integrates smartphone app technology, social support and the outdoor physical environment | To improve aerobic and muscular fitness among adults at risk of, or diagnosed with T2D | Randomized controlled trial, with two parallel groups | wait list/delayed intervention | 20 weeks | To improve aerobic and muscular fitness among adults at risk of, or diagnosed with T2D | Web-based app used required presence of internet; Short duration; Multiple components – difficult to identify active element; Gender biased-female dominant; potential contamination in wait list control group. |
|  | [Poppe et al., 2019](https://pubmed.ncbi.nlm.nih.gov/31376274/) | Fully automated electronic health (eHealth) and mobile health (mHealth) intervention targeting physical activity (PA) and sedentary behaviour (SB) based on the Health Action Process Approach (HAPA)- - MyPlan 2.0 app | To promote behavioural change – sedentary behaviour and Physical activity | Randomised controlled trial, with waitlist control group – (2 intervention + 1 control groups) | Wait list/delayed intervention | 5 weeks | To promote behavioural change – sedentary behaviour and Physical activity | Small sample and no power calculation; Lack of true control/placebo; Not certain whether the detected intervention effects were actually caused by the active ingredients of the intervention eg Phone calls made to intervene negative effects might influence participants’ engagement; Being placed in the waiting-list control group might have influenced behaviour |
| Cardiovascular  (n = 11) | [Barnason et al., 2019](https://europepmc.org/backend/ptpmcrender.fcgi?accid=PMC6453725&blobtype=pdf) | Weight loss telehealth intervention – educational modules + telecoaching + | Promoting weight loss to cardiac revascularization (CR) patients | Randomised controlled trial with two parallel groups (pilot) | Usual care | 12 weeks | To determine if an intervention improves outcomes for overweight and obese CR patients | Small sample size (pilot); Recruitment bias – not ethnic and economic diverse; Unreliability of self-reported measures |
|  | [Duan et al., 2018](https://www.ncbi.nlm.nih.gov/pmc/articles/PMC6277829/) | Web-based Behaviour intervention for lifestyle changes (based on HAPA theory) with monitoring functionality | Improve self-efficacy; Improve Quality of life; Improve Physical Activity and Improve dietary intake (Fruit & Vegetable) | Randomised controlled trial, with waitlist control group | waitlist/delayed intervention | 8 weeks | To evaluate the effect of intervention on physical activity (PA), fruit and vegetable consumption (FVC), lifestyle changes, social-cognitive outcomes, and health outcomes in in Chinese cardiac patients. | Small sample - underpowered;Unreliability of self-reported outcome measures; Short duration of the study and follow up; Recruitment bias – only those with access to computer and internet; Monetary incentives given |
|  | [Eyles et al., 2017](https://pubmed.ncbi.nlm.nih.gov/28631933/) | Innovative smartphone app (SaltSwitch) for scanning food package for salt content and recommends of low salt alternative choices | Support people with cardiovascular disease to make lower salt food choices and Reducing dietary salt consumption | Randomised controlled trial with two parallel groups | usual care | 6 weeks | To determine the effectiveness of app to support people with cardiovascular disease to make lower salt food choices | Inadequate sample size - Low recruitment rate; Low response rate/usage of app |
|  | [Kario et al., 2021](https://www.ncbi.nlm.nih.gov/pmc/articles/PMC8678748/) | A novel digital therapeutic software system including patients’ mobile app linked with web app for health care provider (the HERB) for hypertension management | Hypertension control through lifestyle modification | Randomized controlled trial with two parallel groups. open‐label, multicenter (Pilot) | Active control | 24 weeks | To assess the efficacy of the HERB software system, a smartphone app to treat hypertension in addition to standard guideline-based lifestyle modifications | Unreliability ABPM to accurately measure BP (tends to be affected by user's daily activity); Small sample given the heterogeneity of the baseline characteristics between groups (pilot) |
|  | [Nolan et al., 2018](https://pubmed.ncbi.nlm.nih.gov/30006474/) | E-Counselling on lifestyle ad behaviour change using multimedia and interactive tools | To increase motivation and skill for self-care (exercise, diet, medication adherence, and smoking cessation) for BP control | Randomised controlled trial with two parallel groups – double blind, multicenter | Active control | 56 weeks (12 months) | To evaluate the therapeutic benefit of an intervention on lifestyle ad behaviour change | Recruitment bias – predominantly Caucasian, educated; High attrition rate |
|  | [Tang et al., 2018](https://pubmed.ncbi.nlm.nih.gov/29777560/) | WhatsApp as an information-sharing tool for care | To Improve general knowledge of coronary artery disease risk factors and adherence to a healthy lifestyle | Randomised controlled trial with two parallel groups, open label study | Usual care | 4 weeks | To determine the effect of an intervention on coronary artery disease patient knowledge of and adherence to a healthy lifestyle. | Recruitment bias – convenient sampling; Small sample size - underpowered; Short duration of intervention; Reliability of information delivered were questionable; Since the study was open label (not in controlled environment) the participants were able to obtain information from other sources and confound evaluation |
|  | [Thatthong et al., 2019](https://link.springer.com/article/10.1007/s10389-019-01028-w) | Innovative nutrition educational tool = providing sodium reduction counselling program key messages from the innovative technology (LINE) on smartphones | To improve knowledge, BP level, and urine sodium excretion in at-risk Thai people with hypertension. | Randomised controlled trial with two parallel groups | Active control/Alternative intervention | 8 weeks | To examine the impact of intervention on knowledge, BP level, and urine sodium excretion in at-risk Thai people with hypertension. | Inadequate sample size due to resource constrain; Technical limitations of sodium measurement and sodium database for calculating the sodium intake from dietary recall |
|  | [Tobe et al., 2019](https://www.ncbi.nlm.nih.gov/pmc/articles/PMC8030493/) | Healthy lifestyle and behaviour changes short message service (SMS) through mobile application linked via Bluetooth to MP measuring device. | To improve hypertension awareness, treatment, and control, in remote and vulnerable patient populations | Randomised controlled trial with two parallel groups -Multicenter double blind | Active control | 56 weeks (1 year) | To assess the effect of active (with hypertension specific management SMS) or passive (health behaviors SMS alone) on the difference in blood pressure (BP) reduction | Inadequate sample due to under recruitment |
|  | [Vogel et al., 2017](https://www.ncbi.nlm.nih.gov/pmc/articles/PMC5636132/) | Smart wearable Digital self-tracking device for monitoring Leisure time physical activity | Supporting behavioural change - Improving physical activities among patients undergoing cardiac rehabilitation | Randomised control field experiment study with two parallel groups | Active control | 12 weeks | To investigate the effects of using smart wearables by patients undergoing cardiac rehabilitation on physiological performance | No activity data was generated or recorded by the control group hence No between groups comparison. Instead, each group was compared by performance parameters. physical activity or intensity levels were compared by indicators permitting conclusions to be drawn about physical activities; Longer periods of monitoring may provide an additional insight; Results may not be generalizable for patients with CVD who do not undergo cardiac rehabilitation; Findings of the field experiment might not be transferable to all cardiac patients because of the variety of possible disease symptoms, patterns and medical histories - Personal anamneses were not considered during recruitment; Results are only representative for patients undergoing cardiac rehabilitation at an outpatient rehabilitation centre and hence are not in a stationary rehabilitation setting |
|  | [Widmer et al., 2017](https://www.sciencedirect.com/science/article/pii/S0002870317300510?via%3Dihub) | An online and smartphone-based platform for to support cardiac rehabilitation (providing educational health information and self-reporting patients' healthy lifestyle) | To promote healthy lifestyle | Randomized controlled trial with two parallel groups | Usual care | 12 weeks | To determine whether the intervention administered during cardiac rehabilitation (CR) would reduce CV-related emergency department (ED) visits and rehospitalizations | Relatively a small, single-centre study from a large, tertiary care centre; Inherent difficulty in demonstrating improvement in CVD risk factors and outcomes over optimal medical therapy in a tertiary care CR rehab setting |
|  | [Wong et al., 2020](https://sigmapubs.onlinelibrary.wiley.com/doi/abs/10.1111/wvn.12456) | Web-based educational support intervention (eHES) with educational sessions and recording of health measures + physical exercise | To improve their total physical exercise, self-efficacy for exercise, and cardiovascular risk factor profile | Randomised controlled trial with two parallel groups | Usual care | 24 weeks (6 months) | To examine the effect of this eHES intervention on patients with CHD in terms of their (1) total exercise amount; (2) self-efficacy for exercise; and (3) CVD risk markers. | The intervener and the participants knew the allocation of the groupings; thus, the intervener or Hawthorne effect might have been present in the study; Unreliability of self-reports measures on self-efficacy level and exercise record |
| Multiconditions  (n = 3 ) | [Baer et al., 2020](https://www.ncbi.nlm.nih.gov/pmc/articles/PMC7610192/) | Online weight management program BMIQ (Intellihealth Inc) accessed via a computer, tablet, or smartphone and has patient and professional interfaces. Include educational materials and tracking food and physical activities | To manage weight through nutrition and behavioural change educational | Randomized controlled trial with three parallel groups, multicentre (2 interventions + 1 control) | Usual care | 56 weeks (12 months) | To examine the impact of integrating online weight management program plus population health management on weight management | Samples were not equal across the 3 groups and there were some imbalances in characteristics across the groups; There was heterogeneity in the population health management component of the intervention (due to pragmatic nature of trial), as well as limited data on fidelity to the outreach protocol, making it difficult to determine which components of the intervention worked best; Primary care physicians were minimally involved, which may have affected clinical outcomes and patient satisfaction; Recruitment bias limiting generalizability - the majority of participants were White, well educated, and English-speaking. |
|  | [Chiang et al., 2020](https://pubmed.ncbi.nlm.nih.gov/32511110/#:~:text=Conclusions%3A%20A%2012-week%20home-based%20telehealth%20exercise%20training%20program,quality%20of%20life%20in%20patients%20with%20cardiometabolic%20multimorbidity.) | Home-based telehealth exercise training program | To increase functional capacity and health related quality of life | Randomised control trial with two parallel groups | Usual care | 12 weeks | To determine the effectiveness of an intervention on physical activity, exercise capacity and health-related quality of life in patients with cardiometabolic multimorbidity. | Short-term (12-week) follow-up evaluation; Selection bias caused by the convenience sampling method used; Unreliability of self-report measures used or quantifying PA and health-related quality of life; Important mediators such as self-efficacy, motivation, habit formation, and family or peer support that may influence outcome were not evaluated |
|  | [Taylor et al., 2020](https://pubmed.ncbi.nlm.nih.gov/33243368/) | Web-based behavioural support program for physical activities with pedometer and web-based educational support (-coachER) | To augment exercise referral scheme increase physical activity in inactive individuals with chronic health conditions | Randomised controlled trial with two parallel groups - pragmatic, multicentre | Active control (usual exercise referral scheme) | 56 weeks (12 months) | To determine the impact of the intervention in increasing physical activity | High attrition; Low compliance to intervention (insufficient device wear time) |
