# Supplementary material for: Methodological approaches and author-reported limitations in evaluation studies of digital health technologies (DHT): A scoping review of DHT interventions for cancer, diabetes mellitus, and cardiovascular diseases
Source: PLOS Digit Health. 2025 Apr 24;4(4):e0000806. doi: 10.1371/journal.pdig.0000806 (PMC12021190; doi:10.1371/journal.pdig.0000806)
Supplement: S3 File — (DOCX) [file pdig.0000806.s003.docx]

**Data extraction framework**

| **Data** | **Data description** |
| --- | --- |
| Year | Year that the article was published |
| Country | Name of the country that the work was carried out |
| Name of Condition investigated | Name of the condition investigated   1. *Diabetes* 2. *Cancer* 3. *Cardiovascular* |
| Aim of the DHT intervention | 1. Treatment and therapies 2. Disease self-management 3. Preventive, behaviour change |
| Aim of the study | Aim of the study |
| Outcome measures | 1. Usability 2. Acceptability 3. Disease specific symptoms/measures 4. Physical functioning/activities 5. Psychological change (mood, anxiety, stress, depression, cognitive) 6. Behavioural and lifestyle changes 7. Quality of life 8. Care response efficiency, satisfaction 9. Feasibility |
| Study design | 1. RCT 2. Prospective follow up/Before and after single arm trial 3. Controlled, non-randomised 4. Cross-sectional 5. Retrospective |
| Randomisation status | 1. Not controlled 2. Randomised controlled 3. Controlled, not randomised |
| Comparator type | 1. Usual care 2. Alternative intervention (active control) 3. Historic control 4. Healthy population group as a control 5. Non-responders as control 6. No control |
| Duration of the intervention | Duration of the intervention in week |
| Sample size | Number of participants in the study groups |
| Limitation | Methodological limitations reported by authors |
