# Supplementary material for: Methodological approaches and author-reported limitations in evaluation studies of digital health technologies (DHT): A scoping review of DHT interventions for cancer, diabetes mellitus, and cardiovascular diseases
Source: PLOS Digit Health. 2025 Apr 24;4(4):e0000806. doi: 10.1371/journal.pdig.0000806 (PMC12021190; doi:10.1371/journal.pdig.0000806)
Supplement: S4 File — (DOCX) [file pdig.0000806.s004.docx]

**S4 File: Methodological limitations reported in pilot trials (n = 14) presented according to aim of the DHT intervention, condition investigated and a list of studies in which they appear**

|  | ***Limitations*** | ***DH for treatment/therapy (n = 6)*** | ***DH for self-management***  ***(n = 4)*** | ***DH for prevention***  ***(n = 4)*** | ***Total***  ***n (%)*** |
| --- | --- | --- | --- | --- | --- |
| *1* | *Inadequate sample size due to under recruitment* | *[87], [135]* | *[97]* | *[47], [32], [43]* | *6 (43%)* |
| *2* | *Inadequate sample due to high attrition* | *[37]* |  |  | *1 (7%)* |
| *3* | *Bias in selection and recruitment of participants – self-selection, volunteering, motivated patients* | *[37], [38], [96]* | *[40]* | *[32]* | *5 (36%)* |
| *4* | *Short duration of the study/follow-up* | *[132]* | *[128]* | *[47]* | *3 (21%)* |
| *5* | *Inability to control study environment or group exposure to intervention (contamination)* | *[38]* | *[55], [40]* |  | *3 (21%)* |
| *6* | *Unreliability/validity of measurement tools* | *[37], [38]* | *[40]* | *[47], [32]* | *5 (36%)* |
| *7* | *Inherent systemic difference between study groups* | *[135]* | *[128]* |  | *2 (14%)* |
| *8* | *Issues with intervention compliance and missing data* | *[37]* | *[97]* | *[142], [43]* | *4 (29%)* |
