# Supplementary material for: Methodological approaches and author-reported limitations in evaluation studies of digital health technologies (DHT): A scoping review of DHT interventions for cancer, diabetes mellitus, and cardiovascular diseases
Source: PLOS Digit Health. 2025 Apr 24;4(4):e0000806. doi: 10.1371/journal.pdig.0000806 (PMC12021190; doi:10.1371/journal.pdig.0000806)
Supplement: S5 File — (DOCX) [file pdig.0000806.s005.docx]

**S5 File**

**Methodological limitations reported in full comparative trials evaluating DHT interventions for behaviour change and disease prevention (n = 24), presented according to the condition investigated and a list of studies in which they appear**

| **Limitations** | **Cancer**  **(n = 8)** | **%** | **Diabetes**  **(n = 5)** | **%** | **CVD**  **(n = 8)** | **%** | **Multi**  **(n = 3)** | **%** | **Total (%),**  **n = 24** |
| --- | --- | --- | --- | --- | --- | --- | --- | --- | --- |
| Small sample due under recruitment; no power calculation | [41], [56], [80] | 38% | [59], [93] | 40% | [32], [51], [101], [103], [107], [117] | 75% | [42] | 33% | 12 (50%) |
| Small sample due to high attrition rate |  |  |  |  | [88] | 13% | [102] | 33% | 2 (8%) |
| Bias in selection and recruitment of participants – self-selection, volunteering, motivated patients | [69], [118] | 25% | [60], [77] | 40% | [32], [88], [101], [111] | 50% | [30], [42] | 67% | 10 (42%) |
| Short duration of the study | [41] | 13% | [59], [92], [93] | 60% | [101] | 13% | [42] | 33% | 6 (25%) |
| Inability to control study environment or group exposure to intervention | [118] | 13% | [92] [93] | 40% | [101] | 13% | [30] | 33% | 5 (21%) |
| Unreliability/validity of measurement tools questionable | [41], [108], [69], [118] | 50% | [77] | 20% | [103], [117], [32], [101] | 50% | [42] | 33% | 10 (42%) |
| Unstandardised study procedures/changes in protocol/technology, technical errors |  |  |  |  | [111] | 13% |  |  | 1 (4%) |
| Expert/researcher influence on the outcome | [80] | 13% | [93] | 20% | [120] | 13% | [30] | 33% | 4 (17%) |
| Inherent differences in comparator groups | [108] | 13% |  |  |  |  |  |  | 1 (4%) |
| Confounders not accounted for in evaluation |  |  |  |  | [101] | 13% | [42] | 33% | 2 (8%) |
| Adherence to intervention low/not assessed | [108] | 13% |  |  | [51] | 13% | [102] | 33% | 3 (13%) |
| Multi-component intervention – difficult evaluating individual impact |  |  | [77], [92], [93] | 60% |  |  | [30] | 33% | 4 (17%) |

**Methodological limitations reported in full comparative trials evaluating DHT interventions for treatment and therapies (n = 39) presented according to the condition investigated and a list of studies in which they appear**

| **Limitations** | **Cancer**  **(n = 18)** | **% of Cancer** | **Diabetes**  **(n = 8)** | **% of diabetes** | **CVD**  **(n = 13)** | **% of CVD** | **Total**  **(n = 39)** |
| --- | --- | --- | --- | --- | --- | --- | --- |
| Small sample due to under recruitment | [35], [58], [82], [114] | 22% | [36], [49] | 25% | [74], [81], [85], [116] | 31% | 10 (26%) |
| Small sample due to high attrition rate |  |  | [26] | 13% |  |  | 1(3%) |
| Bias in selection and recruitment of participants – self-selection, volunteering, motivated patients | [86], [125], [58], [61], [62], [142] [114], [115] | 44% | [66], [67], [113] | 38% | [65], [68], [73], [116] | 31% | 15 (38%) |
| Short duration of the study | [24, 29, 46, 125] [64] | 28% | [113] | 13% | [65], [91] | 15% | 8 (21%) |
| Inability to control study environment or group exposure to intervention (contamination) | [79], [125] | 11% | [36] | 13% |  |  | 3 (8%) |
| Unreliability/validity of measurement tools | [29], [61], [86], [112], [125] | 28% | [67] | 13% | [68] | 8% | 7 (18%) |
| Unstandardised study procedures/changes in protocol/technical faults | [109], [114] | 11% | [36] | 13% | [84] | 8% | 4 (10%) |
| Expert/researcher influence on the outcome | [46] [24], [112] | 17% |  |  | [78] [68] | 15% | 5 (13%) |
| Inherent systemic differences between study groups | [79] | 6 % |  |  | [104] | 8% | 2 (5%) |
| Confounders not accounted for in evaluation | [61], [62], [115] | 17% | [52], [66], [95] | 38% | [68], [104] | 15% | 8 (21%) |
| Low adherence to the intervention | [64], [109], [115] | 17% | [26] | 13% | [105] | 8% | 5 (13%) |
| Incomplete or missing data/low adherence to protocol |  |  | [49] | 13% |  |  | 1 (3%) |

**Methodological limitations reported in full comparative trials evaluating DHT interventions for disease self-management (n = 41) presented according to the condition investigated and a list of studies in which they appear**

| **Limitations** | **Cancer**  **(n = 9)** | **%** | **Diabetes**  **(n = 20)** | **%** | **CVD**  **(n = 8)** | **%** | **Multi conditions**  **(n = 4)** | **%** | **Total (%)**  **(n = 41)** |
| --- | --- | --- | --- | --- | --- | --- | --- | --- | --- |
| Small sample due under recruitment; no power calculation | [48], [57] | 22% | [33, 34], [45], [53], [63], [75], [99], [122], [127] | 45% | [119] | 13% |  |  | 12 (27%) |
| Small sample due to high attrition rate |  |  | [123] | 5% |  |  |  |  | 1 (2%) |
| Bias in selection and recruitment of participants – self-selection, volunteering, motivated patients | [25] [57], [100] | 33% | [39], [45], [71], [75], [123] | 25% | [50], [90], [106], [119] | 50% | [28], [76] [89] | 75% | 15 (37%) |
| Short duration of the study | [57] | 11% | [33, 34], [53], [63], [71], [127] | 30% | [124] | 13% | [89], [121]; | 50% | 10 (24%) |
| Inability to control study environment or group exposure to intervention |  |  | [39], [45], [54], [75] | 20% |  |  | [76] | 25% | 5 (12%) |
| Unreliability/Validity of measurement tools questionable |  |  | [54], [123] | 10% | [90], [106], [124] | 38% | [121] | 25% | 6 (15%) |
| Unstandardised study procedures/changes in protocol/technology, technical errors |  |  |  |  | [126] | 13% |  |  | 1 (2%) |
| Expert/researcher influence on the outcome | [25, 48] | 22% | [75] | 5% |  |  | [28], [76] | 50% | 5 (12%) |
| Confounders not accounted for in evaluation | [48] | 11% | [33, 34], [71], [98] | 20% | [106], [126] | 25% | [89], [121] | 50% | 9 (22%) |
| Adherence to intervention low/not assessed |  |  | [33, 34], [54], [75], [127], [143] | 30% | [119],[126] | 25% | [76] | 25% | 9 (22%) |
| Incomplete/missing data/low adherence to protocol |  |  | [122] | 5% | [106], [124] | 25% |  |  | 3 (7%) |
| Multi-component intervention – difficult evaluating individual impact | [25] | 11% | [143], [127] | 10% |  |  | [28] | 25% | 4 (10%) |
